# Association between eating behaviours and food and beverage consumption in male and female children aged 3–6 years: The CORALS cohort

**DOI:** 10.1007/s00394-025-03848-x

**Published:** 2026-01-16

**Authors:** Ivie Maneschy, Ana Daniela Ortega-Ramirez, Andrea Jimeno-Martínez, Mercedes Gil-Campos, Rosaura Leis, Nancy Babio, Santiago Navas-Carretero, Belén Pastor-Villaescusa, Rocío Vázquez-Cobela, Jordi Salas-Salvadó, Pilar De Miguel-Etayo, José Alfredo Martínez, Francisco Jesús Llorente-Cantarero, Rosaura Picáns-Leis, Natalia Ferre Pallas, Azahara I. Rupérez, José Manuel Jurado-Castro, María José de la Torre-Aguilar, Irina Gheorghita, Luis A. Moreno, María L. Miguel-Berges

**Affiliations:** 1 Growth, Exercise, NUtrition and Development (GENUD-B34_23R) Research Group. Instituto Agroalimentario de Aragón (IA2), Universidad de Zaragoza and Instituto de Investigación Sanitaria de Aragón (IIS Aragón), Zaragoza, Spain; 2 Centro de Investigación Biomédica en Red de Fisiopatología de la Obesidad y Nutrición (CIBERObn), Instituto de Salud Carlos III, Madrid, Spain; 3 Faculty of Medicine, University of Colima, Av. Universidad 33 Colonia Las Viboras, 28010 Colima, CP Mexico; 4 Metabolism and Investigation Unit, Maimónides Institute of Biomedicine Research of Córdoba (IMIBIC), Reina Sofia University Hospital, University of Córdoba, 14004 Córdoba, Spain; 5 Primary Care Interventions to Prevent Maternal and Child Chronic Diseases of Perinatal and Developmental Origin (RICORS, RD21/0012/0008); Spanish Network in Maternal, Neonatal, Child and Developmental Health Research (RICORS-SAMID, RD24/0013/0007), Instituto de Salud Carlos III, Madrid, Spain; 6 Servicio de Neonatología, Instituto de Investigación Sanitaria de Santiago (IDIS), Hospital Clínico Universitario de Santiago, Santiago de Compostela, España; 7 Unit of Pediatric Gastroenterology, Hepatology and Nutrition-Pediatrics Service. Hospital, Clínico Universitario de Santiago, Santiago de Compostela, Spain; 8 Pediatrics Nutrition Research Group-Health Research Institute of Santiago (IDIS)-ISCIII, Unit of Investigation in Nutrition, Growth and Human Development of Galicia (GALINUT), University of Santiago de Compostela, Santiago de Compostela, Spain; 9 Grup ANUT-DSM, Departament de Bioquimica i Biotecnologia, Universitat Rovira i Virgili, Unitat de Nutrició Humana, Reus, Spain; 10 Institut d’Investigació Sanitària Pere Virgili (IISPV), Reus, Spain; 11 Department of Nutrition Food Science & Physiology, University of Navarra, 31008 Pamplona, Spain; 12 Center for Nutrition Research, University of Navarra, 31008 Pamplona, Spain; 13 IdisNA, Navarra Institute for Health Research, Pamplona, Spain; 14 Precision Nutrition and Cardiometabolic Health Program, IMDEA-Food Institute (Madrid Institute for Advanced Studies), CEI UAM + CSIC, Madrid, Spain; 15 INstittute of Endocrinology and Nutrition, University of Valladolid, Valladolid, Spain; 16 Pediatrics, Nutrition and Development Research Unit (PediNuR), Universitat Rovira i Virgili, Reus, Spain

**Keywords:** Eating behaviours, Child, Dietary intake, Foods consumption and beverages consumption

## Abstract

**Purpose:**

Eating behaviours influence food intake and long-term eating habits. This study aimed to assess the association between specific eating behaviour traits and the consumption of food and beverage in children aged 3 to 6 years, based on data from the CORAL study.

**Methods:**

Data were obtained from the Spanish CORAL study, a 10-year longitudinal cohort including 1407 participants (699 boys, 708 girls; 4.8 ± 1.0 years). Eating behaviours were assessed using the Child Eating Behaviour Questionnaire (CEBQ) and dietary intake was measured using a validated COME-Kids Food and Beverage Frequency Questionnaire. Principal Component Analysis (PCA) was applied to identify dietary patterns. Associations were analysed with multivariable linear regression adjusted for covariates.

**Results:**

In both sexes, higher Enjoyment of Food scores were associated with greater consumption of fish, fruits, vegetables, pulses, whole grains, and lower consumption of sweets. In contrast, higher Food Fussiness scores were associated with lower consumption of fruits, vegetables, fish, and pulses, and higher intake of sweets. PCA revealed five dietary patterns per sex, explaining 36.62% of the variance in boys and 36.07% in girls. Enjoyment of Food and Food Fussiness were the traits most strongly associated with distinct dietary patterns (*p* < 0.001).

**Conclusion:**

Eating behaviour traits are clearly associated with diet quality in early childhood. Enjoyment of Food supports healthier eating, while Food Fussiness may compromise it. These findings may inform early prevention strategies to encourage healthy eating habits and reduce the risk of childhood overweight and obesity.

**Supplementary Information:**

The online version contains supplementary material available at 10.1007/s00394-025-03848-x.

## Introduction

According to the World Health Organization (WHO), the global prevalence of overweight and obesity in children and adolescents has increased significantly in recent decades, reaching 18% in those aged 5 to 19 years. In Spain, the ALADINO 2023 study (ALimentación, Actividad Física, Desarrollo INfantil y Obesidad), part of the Childhood Obesity Surveillance Initiative (COSI), found that 36.1% of the schoolchildren aged 6 to 9 years were living with overweight or obesity, highlighting the urgent need for preventive strategies [1].

Eating behaviours, defined as the way individual eats, begin to develop from birth and are shaped by a combination of biological, environmental and social factors [2–5], and early childhood represents a critical period for the development of eating habits, as behavioural patterns established during this stage may persist into later life [6]. Eating behaviour consists of two main dimensions: food-approach traits like Food Responsiveness and Enjoyment of food, and food-avoidant traits such as Satiety Responsiveness, Food Fussiness. Understanding these traits during early childhood offers an opportunity to promote healthy eating trajectories and prevent nutrition-related problems later in life [7, 8]. Eating behaviours and food intake are recognized as modifiable factors that may influence the development of childhood obesity [9–11]. Children’s eating behaviour traits can influence not only how much food they consume, but also the type and quality of foods they prefer. Despite this, limited research has examined how specific eating behaviour dimensions relate to actual dietary intake in preschool-aged children, particularly in terms of distinct food and beverage groups [12]. Understanding these associations may help identify early behavioural markers of unhealthy eating trajectories [13]. Although the relationship between eating behaviours and weight status has been extensively explored in school-aged children and adolescents, studies focusing specifically on preschool-aged children remain limited [5, 12]. However, recent evidence suggests that eating behaviour traits are already observable and measurable in children under the age of six, and may influence both dietary intake and health outcomes [13–15]. In a recent systematic review conducted by our group, we identified that most studies to date have evaluated associations between broad eating behaviour traits and individual food or beverage groups, but none have examined these associations using multivariate approaches to derive dietary patterns. To our knowledge, no previous study has explored how specific subscales of eating behaviour relate to overall dietary patterns in early childhood using techniques such as Principal Component Analysis (PCA). Our latest review also highlighted potential associations between food approach behaviours and the consumption of energy-dense foods (e.g., snacks, sweets, sugar-sweetened beverages), which may increase the risk of obesity during childhood [12]. These findings underscore the need for studies specifically designed to examine these complex relationships in preschool-aged populations. The present study aims to address these gaps by analysing how individual eating behaviour traits are associated with both specific food group consumption and broader dietary patterns in children aged 3 to 6 years, using data from a large multicentre cohort.

There are a few questionnaires available to assess eating behaviours in children, among which the Children Eating Behaviour Questionnaire (CEBQ) [7] is one of the most widely used. A recent systematic review and meta-analysis, evaluating the association between childhood adiposity and eating behaviour traits using the CEBQ and the Baby Eating Behaviour Questionnaire (BEBQ) tools, concluded that higher scores on food-approach traits and lower scores on food-avoidant were associated with increased risk of childhood overweight and obesity [8]. Similarly, a cross-sectional study involving 484 school-aged children found that higher food-approach scores were positively associated with overweight in children [5]. Although informative, this study focused on children older than the target age group of the present research (3–6 years), reinforcing the need to explore these associations in preschoolers.

A study using the CEBQ in 283 Spanish children identified an association between eating behaviours and the Body Mass Index (BMI). More specifically, children living with overweight or obesity showed higher scores on food approach traits (e.g., Enjoyment of Food, Food Responsiveness) and lower scores on food-avoidant traits (e.g., Satiety Responsiveness, Food Fussiness) to children in the healthy weight category [16].

Eating behaviour traits may influence not only the quantity but also the quality of food intake. A recent systematic review including thirteen studies from nine countries and participants aged 2 to 16 years found that children with food-approach traits consumed more foods high in sugar and fat, as well as fruits and vegetables. Conversely, children with food-avoidant traits generally consumed less food overall, except for snacks [12]. However, only a small proportion of these studies focused specifically on preschool-aged children, leaving a gap in our understanding of how these behavioural traits relate to dietary intake during early childhood. Given that previous research has shown that boys and girls may differ in how they express eating behaviour traits and respond to food-related cues during early childhood [17–19], we conducted sex-stratified analyses to better capture these behavioural patterns and their associations with dietary intake. Based on the identified gaps in the literature, this study aimed to explore how specific eating behaviour traits are associated with food and beverage consumption in children aged 3 to 6 years. Using data from the CORAL study, we examined both individual food group intake and overall dietary patterns derived through PCA. Understanding these associations during early childhood may help inform strategies to promote healthier eating trajectories and reduce the risk of nutrition-related health issues from an early age.

## Materials and methods

### Study design and sample

The CORAL study (Childhood Obesity Risk Assessment Longitudinal Study) is an ongoing prospective multicentre cohort study, performed in 7 cities in Spain (Barcelona, Cordoba, Pamplona, Reus, Santiago de Compostela, Valencia and Zaragoza). Baseline data were collected between March 2019 and June 2021. The study has a planned follow-up of 10 years, and its main objective is to identify the main risk factors influencing the incidence of childhood obesity. Although the protocol has not yet been published in a peer-reviewed journal, the study is registered at ClinicalTrials.gov (ref: NCT06317883), and details are available at https://corals.es. The total CORALS sample is composed of 1509 children with an age range of 3–6 years old (49.2% boys). The present work is limited to a sample of 1407 children, who had completed the CEBQ and Food and beverage frequency questionnaire (COME-Kids F&B-FQ) and provided socio-economic data. The study was approved by the ethical committees of each local institution and was conducted following the ethical guidelines of the 1964 Declaration of Helsinki.). The questionnaires were completed by parents or legal guardians after signing a written informed consent.

## Eating behaviours

Eating behaviours were assessed with the CEBQ. It is a questionnaire filled in by parents about their children’s eating behaviours. It was developed in the United Kingdom [7] and validated in more than 10 countries around the world [20–24], including Spain. The CEBQ is composed of 35 items answered by parents on a Likert-type scale with possible scores from 1 to 5 (1 = Never, 2 = Rarely, 3 = Sometimes, 4 = Often, 5 = Always). These items measured eight subscales, organised by the questionnaire into two domains: food-approach (Enjoyment of Food, Food Responsiveness, Desire to Drink and Emotional Overeating) and food-avoidant (Satiety Responsiveness, Slowness in Eating, Food Fussiness and Emotional Undereating). No total score was calculated; instead, mean scores were computed for each of the eight subscales, in line with the original scoring recommendations. These subscale scores were then used as independent variables in subsequent statistical analyses.

Higher scores on each subscale indicate greater expression of the specific behaviour trait. For example, a higher score in Food Responsiveness reflects stronger sensitivity to external food cues, while a higher score in Food Fussiness indicates more selective or avoidant behaviour toward certain foods. These scores do not inherently represent healthier or unhealthier behaviours but must be interpreted in context with dietary intake and health outcomes.

## Food and beverage consumption

Food and beverage consumption was assessed using the COME-Kids F&B-FQ, a 125-item semi-quantitative food frequency questionnaire (FFQ) designed to estimate detailed and habitual intake over the past year [25]. This instrument was derived from a FFQ previously validated in Spain [26], and has recently been formally validated in a representative sample of Spanish children aged 3 to 11 years, demonstrating acceptable reproducibility and relative validity for assessing food and nutrient intake. Parents or caregivers were asked how often, on average, the child had consumed the specified portion of each item in the past year during face-to-face interviews by trained registered dietitians.

The COME-Kids F&B-FQ has an optically readable form format, which allowed it to be scanned using Evaldara^®^ software. The software automatically exports the reported frequency of consumption for the 125 items, which was incorporated into the e-Diet Base Universitat Rovira I Virgili (URV) software. This software calculates estimated intakes of nutrients and food groups by multiplying the frequency of consumption of each item by the nutrient content of the portion specified in the COME-Kids F&B-FQ.

Food and beverage consumption are expressed in grams per day. In this study, we categorised foods into twenty-three groups according to their nutritional similarity: [1] Dairy products; [2] Dairy desserts; [3] Eggs; [4] Meat; [5] Processed meat; [6] Fish and seafood; [7] Vegetables; [8] Tubers; [9] Fruits; [10] Nuts; [11] Olives; [12] Refined cereals; [13] Whole grains; [14] Pulses; [15] Oils; [16] Sweets; [17] Sugar and cocoa; [18] Snacks; [19] Prepared foods; [20] Sauces; [21] Water; [22] Sugar sweetened beverages (SSB); and [23] Coffee and tea. The categorization of food groups is shown in Supplementary Table 1.

## Socioeconomic status

A set of standardised, self-administered questionnaires including information on sex, age and socio-economic status (SES) was used. In this study, maternal education was used as a proxy for SES, as it is widely considered a stable and reliable indicator of socioeconomic conditions in early childhood, particularly in relation to child nutrition and health outcomes [27, 28]. Maternal educational attainment was first measured in nine categories and then re-categorised into three: Primary education; Middle education; and Higher education.

### Statistical analysis

All analyses were conducted using the Statistical Package for Social Science (SPSS) version 25.0. Graphs were generated in GraphPad Prism version 8.0 and edited in Adobe Illustrator version 2022. Analyses were stratified by sex, based on existing evidence of differences in eating behaviours and dietary intake between boys and girls in early childhood. This decision was made a priori to allow for a more precise characterisation of associations within each sex. Data are presented as mean and standard deviation (SD) for quantitative variables and as percentage (number) for qualitative variables. Differences were assessed using t-tests for continuous variables and chi-squared tests for categorical variables. Linear regression models were first used to analyse the associations between eating behaviours subscales (independent variables) and food and beverage groups (dependent variables). These models provided beta coefficients (β) with 95% confidence intervals (CI) and were adjusted for maternal education, child´s age, centre and total energy intake.

Principal component analysis (PCA) was then used to identify dietary patterns specific to boys and girls. For component extraction, the eigenvalue greater than 1 criterion was used along with varimax rotation to improve interpretability. Following the identification of these dietary patterns, additional linear regression analyses were conducted to assess the associations between the eating behaviours subscales and the identified dietary patterns. These regression models were also adjusted for potential confounding variables such as child´s age, maternal education, socioeconomic status, centre and total energy intake.

Statistical significance was set at *p* < 0.05. Beta coefficients and 95% confidence intervals were used to interpret the strength and direction of associations. Although no formal correction for multiple comparisons was applied, the consistency of associations across models was considered in the interpretation of findings.

## Results

In total, 1,407 children (49.7% boys) were included in the analyses. The descriptive characteristics of the participants can be found in Table 1. The average age was 4.8 ± 1.0 years. Regarding the average consumption of the different food groups, significant differences were found between sexes in dairy products, dairy desserts and sweets and (all *p* < 0.05). Specifically, boys had a higher average intake of dairy products (485.2 ± 283.0 g/day vs. 438.9 ± 240.5 g/day; *p* = 0.038), dairy desserts (86.8  ±  112.8 g/day vs. 77.6  ±  105.8 g/day; *p* = 0.018), and sweets (53.6 ± 40.4 g/day vs. 46.9 ± 36.2 g/day; *p* = 0.001) compared to girls.

After adjusting for maternal education, child’s age, study centre, and total energy intake, we observed several significant associations between CEBQ subscales and food and beverage intake. Notably, in both sexes higher Enjoyment of Food scores were associated with greater consumption of vegetables, fruits, fish, pulses, and whole grains, and lower consumption of sweets. In contrast, higher Food Fussiness scores were linked to lower intake of these nutrient-dense food groups and increased consumption of sweets and sugar. Satiety Responsiveness also showed relevant associations, with higher scores being related to reduced intake of several food groups, including meat and vegetables. These three subscales were highlighted due to the number and consistency of their significant associations across multiple food groups, as well as their relevance in both sexes. In particular, Enjoyment of Food and Food Fussiness consistently emerged as the strongest predictors of intake of across a range of food categories, including nutrient-dense items such as fruits, vegetables, and fish, as well as energy-dense or sugar-rich foods like sweets and desserts.

Supplementary Table 2 shows all the quantitative results of the CEBQ subscales by sex.

**Table 1:** Descriptive characteristics of the children sample from the CORAL study (*n* = 1407)

	Boys (*n* = 699)	Girls (*n* = 708)	*p*-value
Age (years), mean ±SD	4.7 ± 1.0	4.8 ± 1.0	
*City, n (%)*			
Córdoba	208 (29.8)	191 (27.0)	
Santiago de Compostela	119 (17.0)	146 (20.5)	
Reus	134 (19.2)	115 (16.2)	
Zaragoza	103 (14.7)	112 (15.8)	
Pamplona	66 (9.4)	76(10.7)	
Valencia	40 (5.7)	31 (4.4)	
Barcelona	29 (4.1)	38 (5.4)	
*Mother’s Education, n (%)*			
Basic	128 (18.3)	135 (19.1)	
Middle	225 (32.2)	213 (30.1)	0.798
Higher	346 (49.5)	360 (50.8)	
*CEBQ subscales,mean ±SD*			
EF (enjoyment of food)	3.3 ± 0.7	3.4 ± 0.7	0.710
FR (food responsiveness)	2.1 ± 0.9	2.1 ± 0.8	0.663
DD (desire to drink)	2.3 ± 0.9	2.2 ± 0.9	0.956
EOE (emotional overeating)	1.6 ± 0.6	1.6 ± 0.6	0.707
SR (satiety responsiveness)	2.7 ± 0.7	2.8 ± 0.7	0.277
SE (slowness in eating)	2.8 ± 0.8	2.9 ± 0.8	0.135
FF (food fussiness)	2.9 ± 0.8	2.8 ± 0.8	0.766
EUE (emotional undereating)	2.8 ± 0.9	2.8 ± 0.9	0.817
Energy intake, mean ±SD	1798.9 ± 518.1	1718.7 ± 446.3	**< 0.015**
*Food and beverage intake, mean ±SD*			
Dairy products (g/day)	485.2 ± 283.0	438.9 ± 240.5	**0.038**
Dairy desserts (g/day)	86.8 ± 112.8	77.6 ± 105.8	**0.018**
Eggs (g/day)	23.3 ± 10.3	24.8 ± 21.5	1.000
Meat (g/day)	55.7 ± 30.2	56.8 ± 26.8	0.565
Processed meat (g/day)	14.6 ± 10.7	14.9 ± 11.9	0.858
Fish and seafood (g/day)	33.4 ± 21.9	35.1 ± 24.6	0.139
Vegetables (g/day)	79.1 ± 57.7	77.5 ± 59.0	0.653
Tubers (g/day)	37.0 ± 28.4	35.7 ± 19.4	0.998
Fruits (g/day)	185.4 ± 130.7	181.7 ± 123.3	0.887
Nuts (g/day)	3.5 ± 6.4	4.0 ± 6.7	0.526
Olives (g/day)	3.7 ± 5.2	4.3 ± 6.6	0.262
Refined grains (g/day)	70.2 ± 36.9	67.0 ± 34.7	0.196
Whole grains (g/day)	8.3 ± 15.9	8.5 ± 18.6	0.717
Pulses (g/day)	18.5 ± 9.7	18.3 ± 13.7	0.567
Oils (g/day)	27.1 ± 16.8	26.8 ± 16.1	0.524
Sweets (g/day)	53.6 ± 40.4	46.9 ± 36.2	**0.001**
Sugar and cocoa (g/day)	10.4 ± 11.1	10.3 ± 10.1	0.399
Snacks (g/day)	13.2 ± 12.0	13.1 ± 12.0	1.000
Prepared foods (g/day)	24.4 ± 30.8	25.6 ± 31.4	0.967
Sauces (g/day)	2.5 ± 1.8	2.5 ± 1.6	0.233
Water (g/day)	862.4 ± 368.9	874.2 ± 384.8	0.978
Sugar sweetened beverages (g/day)	135.5 ± 169.6	116.4 ± 139.2	0.239
Coffee and tea (g/day)	6.2 ± 26.4	8.2 ± 39.3	1.000

CEBQ, child eating behaviour questionnaire; SD, standard deviations. Bold letters show significant difference between boys and girls in the chi-squared test for categorical variables and t-test for continuous variables (*p* < 0.05). Table 1 presents unadjusted descriptive statistics comparing boys and girls. Statistical comparisons (pvalues)are based on independent t tests for continu ous variables and chi squared tests for categorical variables. Adjustedassociations are presented in subsequent sections of the r esults

Table 1 presents the descriptive characteristics of the study sample, stratified by sex. As the analytical approach in this study was based on sex-stratified associations, we did not compute combined summary values or between-sex comparisons (e.g., p-values) for these variables. This presentation aligns with the study’s design and allows for consistent interpretation of sex-specific findings throughout the results.

**Fig. 1:**
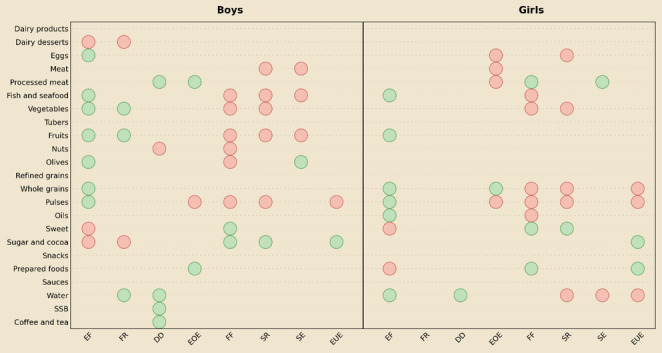
Direction and significance of associations between CEBQ subscales and intake of food and beverage groups in boys and girls. EF, enjoyment of food; FR, food responsiveness; DD, desire to drink; EOE, emotional overeating; FF, food fussiness; SR, satiety responsiveness; LE, slowness in eating; EUE, emotional undereating. Circles represent direction and significance of associations from adjusted linear regression models:green, significant positive association (*p* < 0.05); red, significant negative association (*p* < 0.05)

**Fig. 2:**
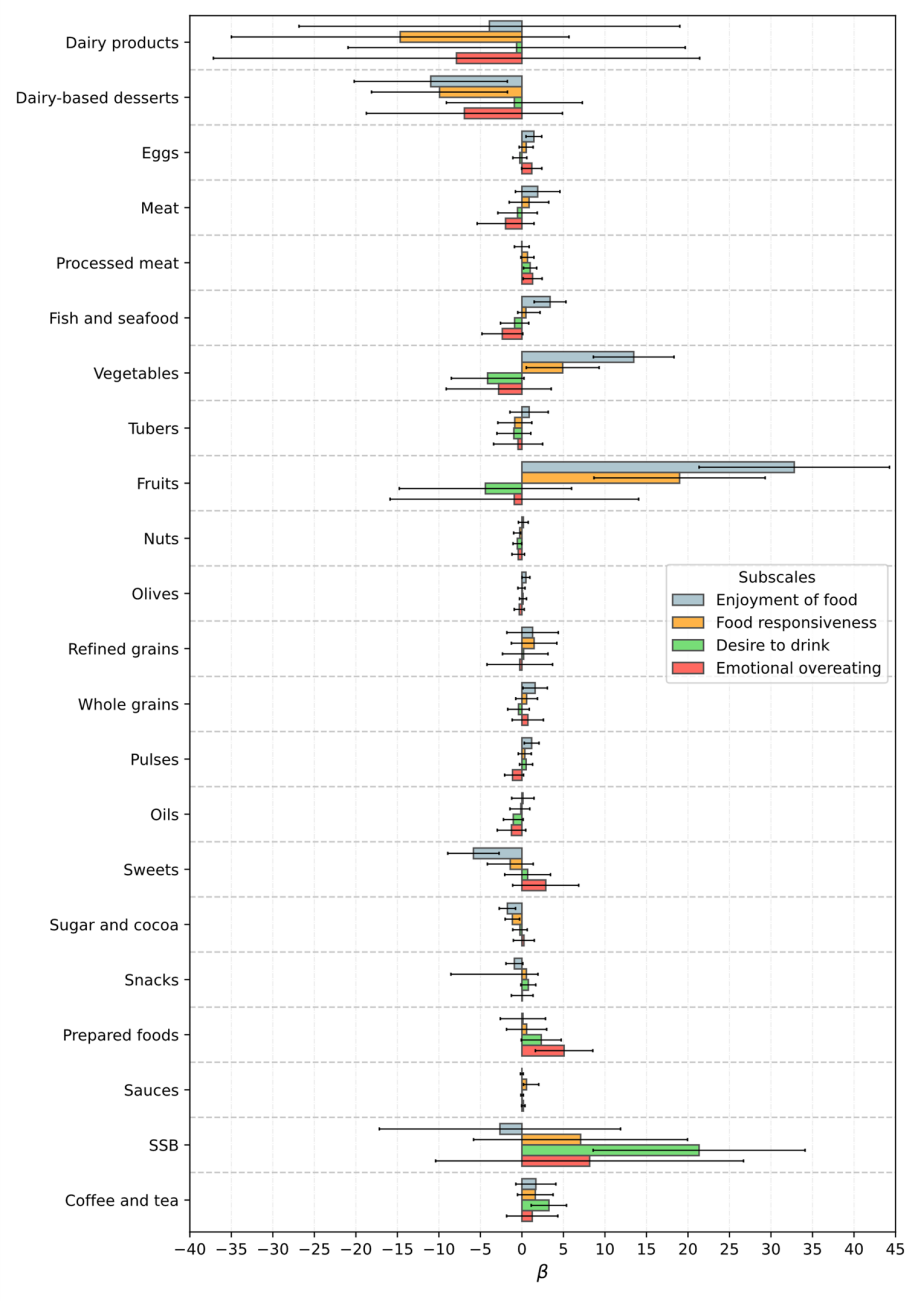
Association, Beta (95% CI), between CEBQ food-approach subscales and food and beverage groups intake, in boys from the CORAL study

**Fig. 3:**
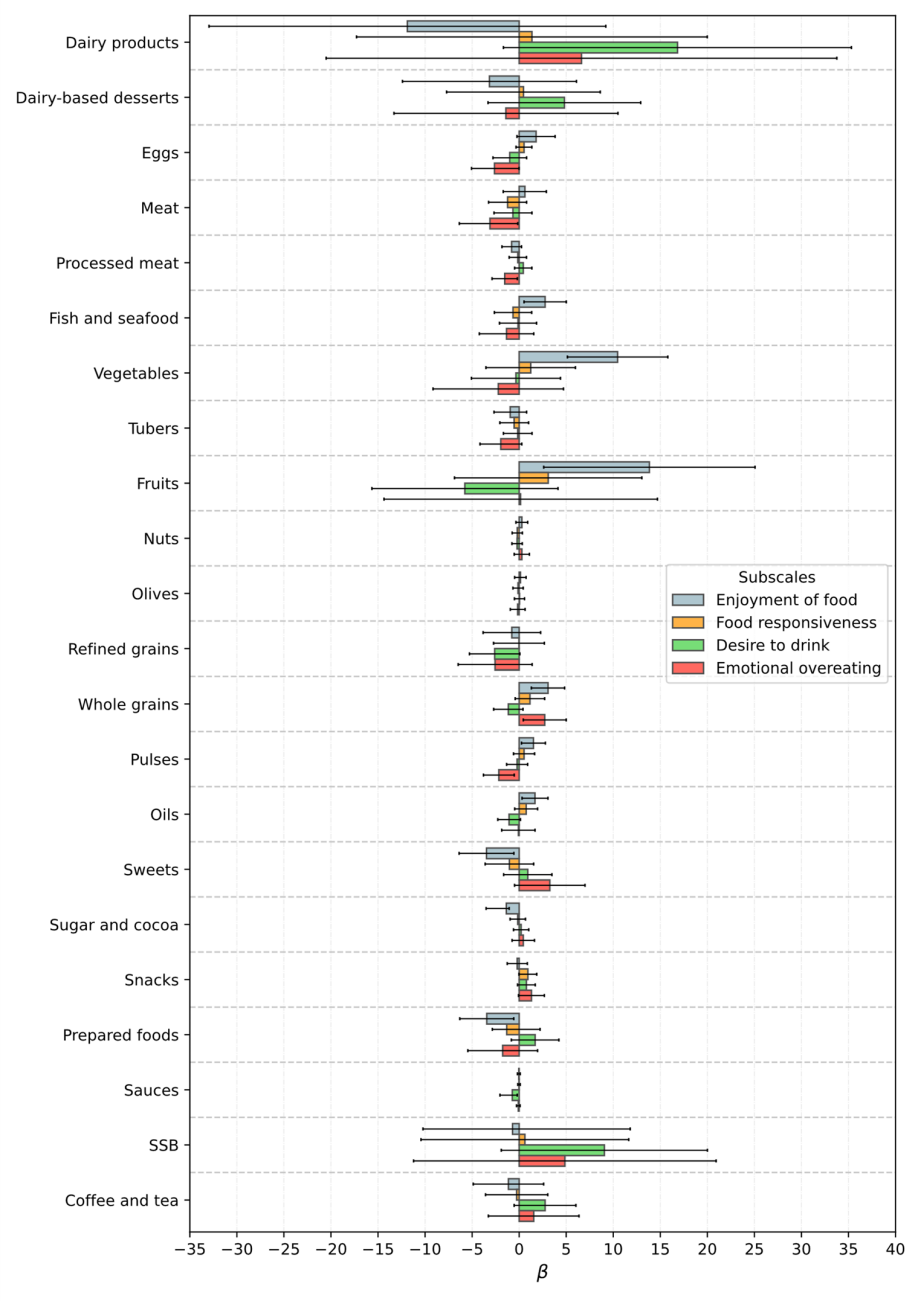
Association, Beta (95% CI), between CEBQ food-approach subscales and food and beverage groups intake, in girls from the CORAL study

To facilitate interpretation of the large number of associations explored, we summarized the direction of statistically significant associations (*p* < 0.05) between CEBQ subscales and the intake of 23 food and beverage groups in a single figure (Fig. 1) separately for boys and girls. This summary complements Figs. 2 and 3 and supports interpretation of the detailed results described below.

Significant associations were observed between food-approach subscales and specific food and beverage groups in both boys and girls (Figs. 2 and 3). In particular, higher Enjoyment of Food scores were associated with greater intake of nutrient-dense foods such as fruits, vegetables, whole grains, pulses, and fish and seafood. For instance, among boys, fruit intake was positively associated with Enjoyment of Food (β = 32.80; 95% CI: 21.33 to 44.26; *p* < 0.001), and sweets consumption was negatively associated (β = − 5.84; 95% CI: − 8.93 to − 2.75; *p* < 0.001). In girls, similar patterns were observed, with additional associations involving higher intake of water and oils, and lower intake of prepared foods. These results suggest that children with higher enjoyment of food tend to follow healthier dietary patterns.

For the Food Responsiveness subscale, significant associations were observed only among boys. Higher scores were associated with greater intake of vegetables (β = 7.36; 95% CI: 2.72 to 12.00; *p* = 0.002) and fruits (β = 15.49; 95% CI: 4.62 to 26.36; *p* = 0.005), and lower intake of sugar and cocoa (β = − 1.10; 95% CI: − 1.94 to − 0.26; *p* = 0.011). This suggests that boys more responsive to food cues might consume more fruits and vegetables, while showing reduced intake of sugary products.

Desire to Drink was positively associated with water intake in both boys (β = 59.56; 95% CI: 28.64 to 90.48; *p* < 0.001) and girls (β = 38.69; 95% CI: 6.37 to 70.98; *p* = 0.019), indicating that this subscale may reflect a preference for fluids in general. However, among boys, it was also associated with higher intake of sugar-sweetened beverages and coffee and tea, and lower intake of nuts, suggesting a broader tendency toward drinkable or liquid-based items, not always aligned with healthy choices.

Emotional Overeating showed different patterns by sex. In boys, higher scores were linked to greater intake of processed meat (β = 2.07; 95% CI: 0.41 to 3.72; *p* = 0.014) and prepared foods, and lower intake of pulses. In girls, higher Emotional Overeating was associated with lower consumption of meat (β = − 3.49; 95% CI: − 6.31 to − 0.66; *p* = 0.016) and processed meat (β = − 1.38; 95% CI: − 2.73 to − 0.03; *p* = 0.045), but showed a positive association with whole grains. These findings may reflect differences in how emotional overeating manifests in early childhood, depending on sex.

**Fig. 4:**
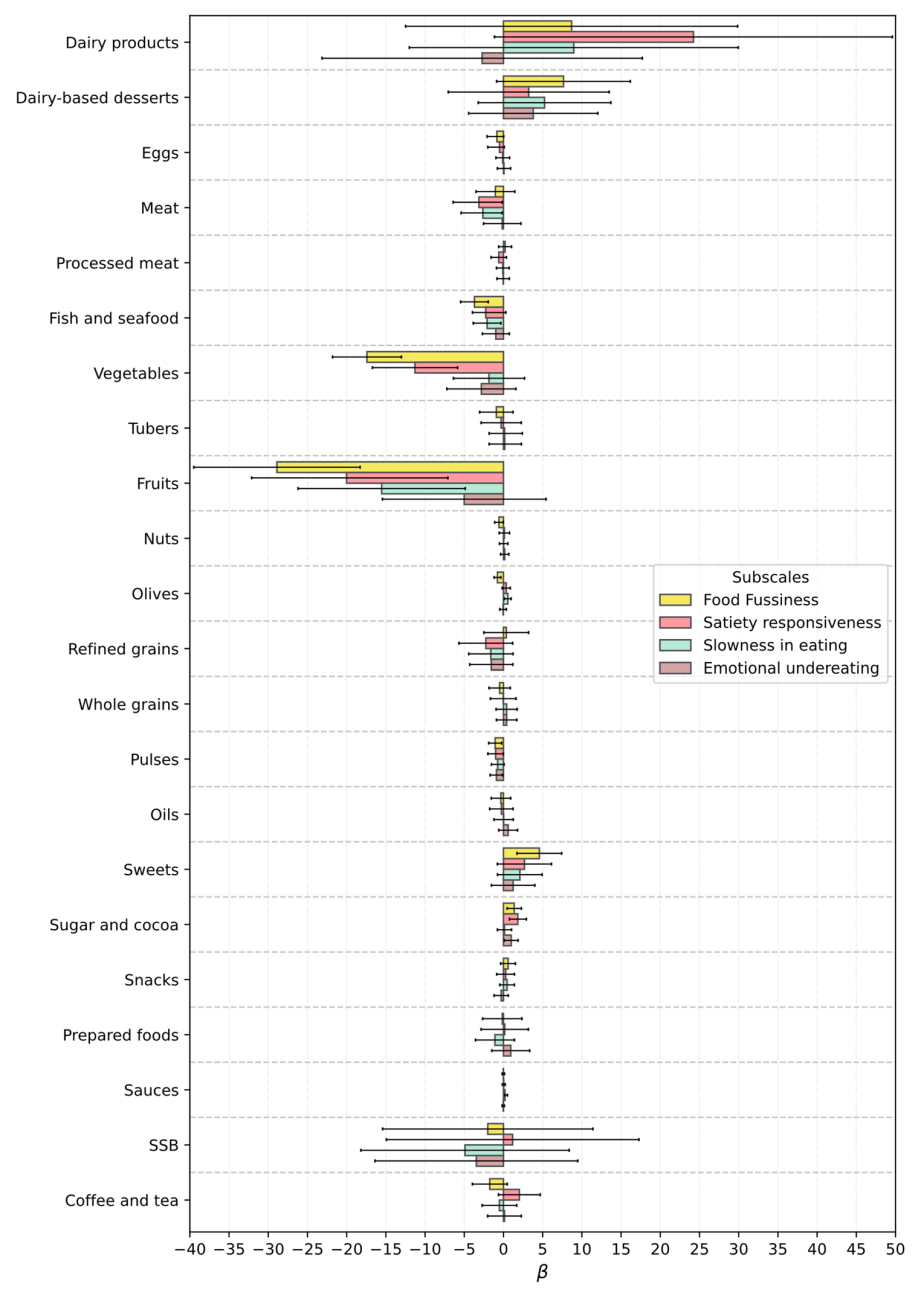
Association, Beta (95% CI), between CEBQ food-avoidant subscales and food and beverage groups intake, in boys from the CORAL study

**Fig. 5:**
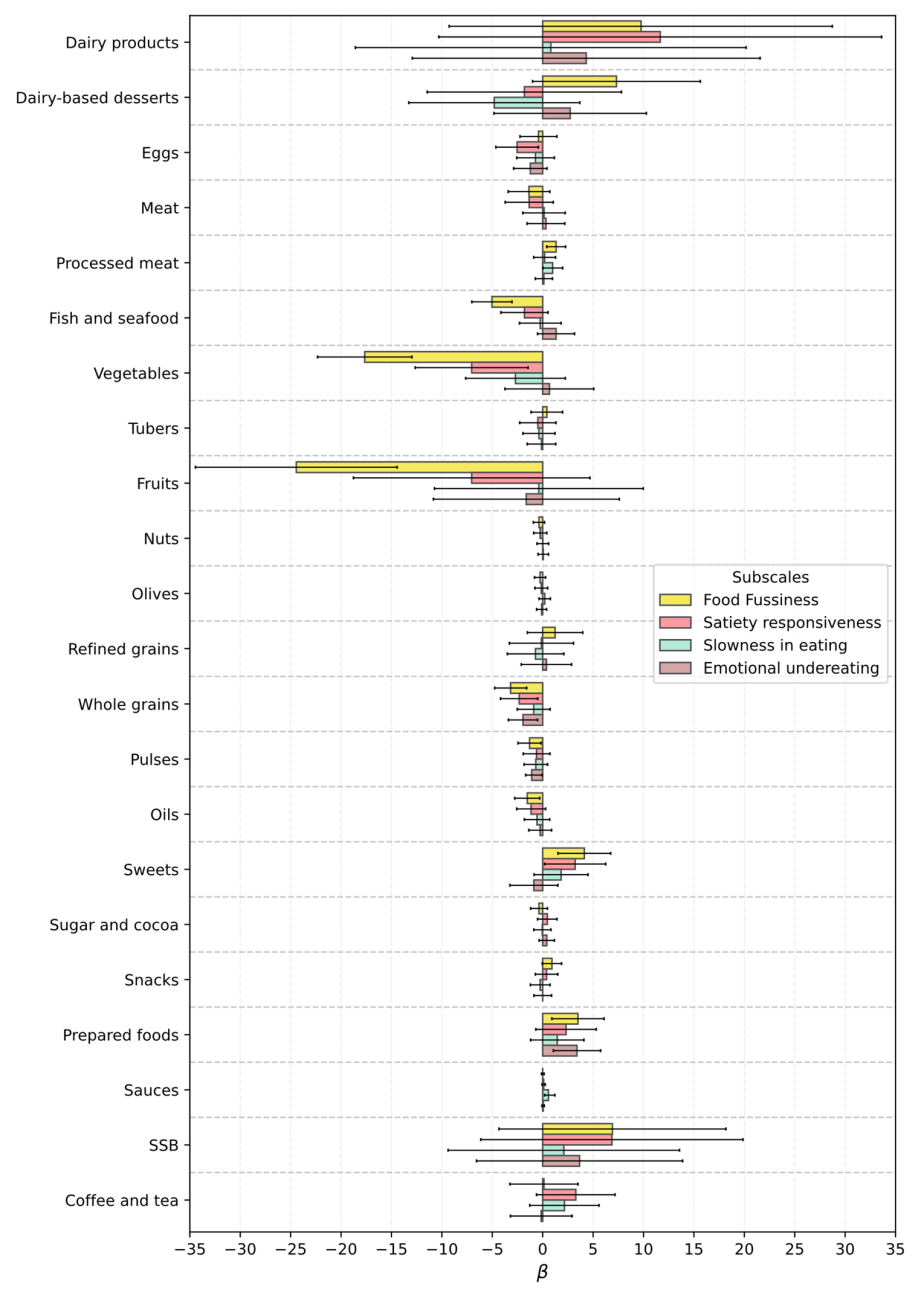
Association, beta (95% CI), between CEBQ food-avoidant subscales and food and beverage groups intake, in girls from the CORAL study

Regarding food-avoidant subscales, the highest number of associations with food intake was observed for Food Fussiness and Satiety Responsiveness (Figs. 4 and 5). Higher Food Fussiness scores were consistently associated with lower consumption of nutrient-dense food. For example, among girls, higher FF was negatively associated with vegetable intake (β = − 17.65; 95% CI: − 22.33 to − 12.97; *p* < 0.001) and fruit intake (β = − 24.44; 95% CI: − 34.45 to − 14.44; *p* < 0.001). These patterns were observed in both sexes and extended to fish, pulses, and whole grains, suggesting that fussier eaters tend to consume fewer nutrient-dense food and a more limited variety overall.

Satiety Responsiveness was also associated with reduced intake across several food groups. Among boys, higher SR scores were linked to lower intake of fruits (β = − 14.09; 95% CI: − 22.15 to − 6.04; *p* = 0.001) and pulses (β = − 0.96; 95% CI: − 1.80 to − 0.12; *p* = 0.025), and higher intake of sugar and cocoa. In girls, negative associations were observed with vegetables, eggs, water, and other core food, while sweets and sugar and cocoa showed positive associations. These results suggest that higher satiety sensitivity may contribute to an overall decrease in dietary variety and preference for sweeter items.

Slowness in Eating showed fewer and more inconsistent associations. Among boys, higher SE scores were associated with lower consumption of meat and fish, while in girls, a positive association was observed with processed meat intake and a negative one with water. These findings indicate that this subscale may capture behaviours related to tempo and mealtime dynamics, rather than clear dietary patterns.

Finally, Emotional Undereating was negatively associated with the intake of pulses in both boys and girls. Among boys, a positive association was observed with sugar and cocoa (β = 0.84; 95% CI: 0.13 to 1.56; *p* = 0.020), while among girls, higher scores were linked to greater consumption of prepared foods and lower intake of whole grains and water. Together, these associations suggest that emotional suppression of appetite in young children may relate to more limited and lower in nutrient-dense food choices.

Principal component analysis identified five distinct dietary patterns in both boys and girls, with sex-specific factor loadings, as indicated in Tables 2 and 3.

For boys, Convenience pattern explained 8.14% of the variance and was characterised by high loadings for processed meats, snacks, SSB and prepared foods. The Refined carbs pattern accounted for 7.72% of the variance and included high loadings on refined grains, sweets and sugar and cocoa, with a negative loading on whole grains. The Animal protein pattern explained 7.31% of the variance and was characterised by high loadings on meat, fish and seafood and refined cereals. The Dairy-starch pattern accounted for 6.95% of the variance, with high loadings on dairy products, dairy desserts and tubers. The Veg & fats pattern explained 6.46% of the variance and included high loadings on vegetables, nuts and oils. The cumulative variance explained by these dietary patterns was 36.62%.

For girls, the Veg-seafood-legumes pattern explained 8.69% of the variance and included high loadings on vegetables, fish and seafood, and pulses. The Meat-convenience pattern explained 7.80% of the variance with high loadings on meat, tubers and prepared foods. The Sugary foods pattern explained 6.81% of the variance and included high loadings on sugar and cocoa and SSB. The Hydration & fats pattern accounted for 6.52% of the variance and was characterised by high loadings on oils and water, with a negative loading for dairy products. The Plant snacks pattern explained 6.23% of the variance with high loadings on nuts, olives and snacks. The cumulative variance explained by these dietary patterns was 36.07%.

**Fig. 6:**
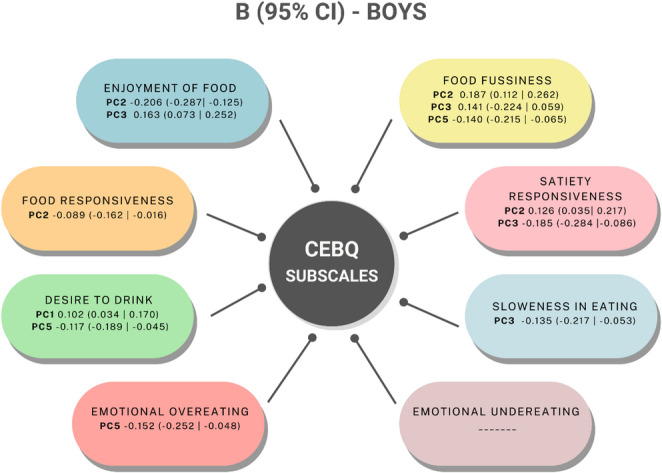
Beta (95% CI) for the association between CEBQ subscales and dietary patterns, in boys from the CORAL study. CEBQ, child eating behaviour questionnaire; CI, confidence intervals; PC, principal component; PC1, convenience pattern; PC2, refined carbs pattern; PC3, animal protein patternpc5; veg and fats pattern. PC, principal component. Pattern names are based on dominant food group loadings (see Table 2)

**Fig. 7:**
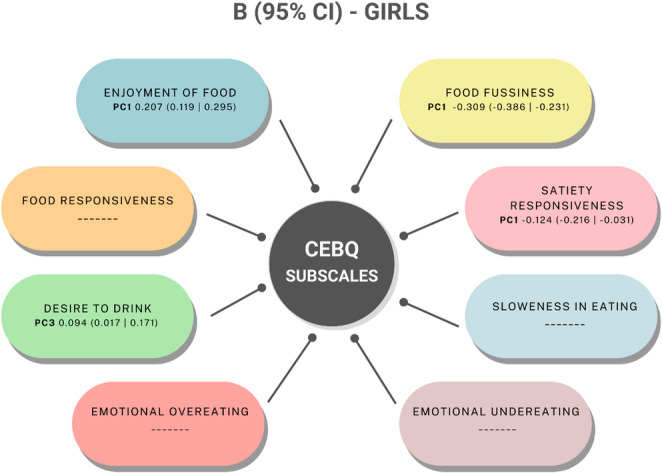
Beta (95% CI) for the association between CEBQ subscales and dietary patterns, in girls from the CORAL study. CEBQ, child eating behavior questionnaire; CI, confidence intervals; PC, principal component. PC1, veg-seafood-legumes pattern; PC3, Sugary foods pattern. PC, principal component. Pattern names are based on dominant food group loadings (see Table 3)

Regression analysis between eating behaviours subscales and dietary patterns is summarized in Figs. 6 and 7, complete information on this analysis can be found in supplementary Table 3. Significant associations were found between the eating behaviours subscales and the identified dietary patterns.

Regarding the Enjoyment of Food subscale, higher scores were negatively associated with the Refined carbs pattern and positively associated with the Animal protein pattern in boys, whereas in girls, it showed a positive association with the Veg-seafood-legumes pattern. Concerning the Food Responsiveness subscale, higher scores were negatively associated with the Refined carbs pattern in boys, but no significant associations were found in girls. The Desire to Drink subscale showed positive associations with the Convenience pattern and negative associations with the Veg & fats pattern in boys; in girls, it was positively associated with the Sugary foods pattern. For the Emotional Overeating subscale, boys showed a negative association with the Veg & fats pattern, whereas no significant associations were observed in girls.

The Food Fussiness subscale was positively associated with the Refined carbs pattern and negatively associated with the Veg & fats pattern in boys; in girls, it was negatively associated with the Veg-seafood-legumes pattern. Finally, the Satiety Responsiveness subscale was positively associated with the Refined carbs pattern and negatively associated with the Animal protein pattern in boys. In girls, a negative association was observed with the Veg-seafood-legumes pattern. These results highlight sex differences in the relationships between eating behaviours and dietary patterns.

**Table 2:** Factor loadings of identified dietary patterns in boys

	Convenience pattern (8.14%)	Refined carbs pattern (7.72%)	Animal protein pattern (7.31%)	Dairy-starch pattern (6.95%)	Veg & fats pattern (6.46%)
Dairy products	− 0.041	**0.362**	− 0.150	**0.467**	0.052
Dairy desserts	**0.332**	0.061	0.033	**0.658**	− 0.119
Eggs	− 0.036	0.026	0.042	− 0.036	0.048
Meat	0.060	0.151	**0.800**	− 0.026	0.029
Processed meat	**0.632**	0.151	0.175	0.147	− 0.122
Fish and seafood	**0.069**	–0.079	0.296	0.057	**0.442**
Vegetables	− 0.044	− 0.417	0.150	− 0.067	**0.492**
Tubers	− 0.043	0.042	0.270	**0.742**	0.028
Fruits	− 0.167	0.200	− 0.105	0.173	**0.668**
Nuts	− 0.060	0.042	0.083	0.048	**0.685**
Olives	**0.466**	− 0.164	0.078	− 0.169	**0.266**
Refined grains	0.061	**0.467**	**0.405**	0.181	0.135
Whole grains	0.026	**− 0.651**	− 0.129	0.196	0.204
Pulses	0.062	− 0.285	0.207	0.226	**0.293**
Oils	**0.198**	0.080	− 0.085	− 0.115	**0.586**
Sweet	0.099	**0.573**	0.186	0.238	− 0.121
Sugar and cocoa	**0.314**	**0.408**	− 0.041	− 0.031	− 0.040
Snacks	**0.589**	0.169	− 0.146	0.232	0.089
Prepared foods	**0.444**	0.159	0.231	0.136	− 0.232
Sauces	0.244	0.250	− 0.094		0.081
Water	− 0.110	− 0.060	0.134	0.104	− 0.066
SSB	**0.593**	− 0.070		0.219	0.067
Coffee and tea	-0.074	− 0.094	0.060	0.095	

Table shows rotated factor loadings from principal component analysis (PCA) for five dietary patterns in boys. Only loadings ≥ |0.30| are considered meaningful and are shown in bold. Patterns were labelled based on dominant food group loadings

**Table 3:** Factor loadings of identified dietary patterns in girls

	Veg seafood legumes pattern (8.69%)	Meat-convenience pattern (7.80%)	Sugary foods pattern (6.81%)	Hydration & fats pattern (6.52%)	Plant snacks pattern (6.23%)
Dairy products	0.200	0.052	0.270	**− 0.450**	− 0.051
Dairy desserts	− 0.092	0.055	– 0.136	0.193	0.193
Eggs	0.048	0.055	0.101	− 0.042	− 0.042
Meat	0.163	**0.634**	0.068	0.088	0.088
Processed meat	− 0.181	0.046	– 0.239	0.176	0.134
Fish and seafood	**0.442**	**0.553**	– 0.072	− 0.133	0.086
Vegetables	**0.758**	− 0.044	– 0.036	0.144	0.144
Tubers	0.028	**0.645**	0.178	0.039	0.133
Fruits	**0.668**	0.082	0.115	0.067	0.067
Nuts	**0.357**	0.119	– 0.179	− 0.062	**0.417**
Olives	0.124	0.072	0.133	0.062	**0.726**
Refined grains	0.135	0.135	0.125	− 0.074	0.317
Whole grains	0.204	− 0.037	– 0.029	− 0.032	0.322
Pulses	**0.509**	0.265	– 0.025	0.081	− 0.140
Oils	0.213	− 0.100	– 0.100	**0.718**	0.065
Sweet	− 0.271	0.178	**0.374**	0.136	0.136
Sugar and cocoa	− 0.041	0.298	**0.630**	− 0.046	0.026
Snacks	− 0.177	0.027	0.198	− 0.056	**0.640**
Prepared foods	− 0.240	**0.598**	− 0.070	0.068	0.060
Sauces		0.179		**0.650**	0.084
Water		0.046	0.173	**0.408**	− 0.184
SSB	0.044		**0.447**	0.221	
Coffee And Tea		− 0.162	0.718		0.203

Table shows rotated factor loadings from principal component analysis (PCA) for five dietary patterns in girls. Only loadings ≥ |0.30| are considered meaningful and are shown in bold. Patterns were labelled based on dominant food group loadings

## Discussion

As dietary habits and behaviours are established early in life, it is important to understand how eating behaviours may influence food intake. Eating behaviours have been suggested as one of the main influencing factors of food and beverage intake; however, there are few studies on the relationship between eating behaviours and foods intake, especially when assessing a large number of food groups. This study assessed the relationship between eating behaviours subscales and 23 foods and beverage groups and dietary patterns derived by using PCA analysis. Eating behaviours subscales that were the main determinants of food intake were Enjoyment of Food (food-approach subscale) and Food Fussiness (food-avoidant subscale), both of which showed stronger associations with specific food groups, such as nutrient-dense or energy dense items, rather than with total intake. In both sexes, Food Fussiness was associated with lower consumption of fruits, vegetables, fish and pulses, and higher intake of sweets; in contrast, Enjoyment of Food, was associated with higher consumption of fish, fruits, vegetables, pulses, whole grains and lower consumption of sweets. To the best of our knowledge, this is the first study assessing the association between eating behaviours and children’s dietary patterns using PCA analysis. Although PCA has not been widely applied in this context, the broader literature on eating behaviours and food consumption in children remains limited. For example, a recent systematic review in children and adolescents identified only 13 studies examining these associations [12], highlighting the fragmented nature of the available evidence.

### Food-approach subscales

The food-approach subscales, specifically Enjoyment of Food, Food Responsiveness, and Desire to Drink, showed positive significant results for several food groups. This was to be expected since they are subscales that influence greater consumption [8, 29, 30]. Among the food-approach subscales, Enjoyment of Food stands out due to the consistency and number of significant associations across multiple nutrient-dense food groups and in both sexes. In fact, Enjoyment of Food was associated with higher consumption of food groups commonly recommended in childhood diets, including fish and seafood, vegetables, fruits, whole grain cereals and pulses in both sexes, as well as eggs in boys and oils in girls; and it was negatively associated with sweets, dairy desserts and prepared foods. These results corroborate those of a recent study [18] showing that children with higher Enjoyment of Food scores have a greater interest in food in general and also greater consumption of the main meals per day. Similarly, Blissett et al. [15]. found that this trait was associated with higher intake of overall food, including chocolate. Another study [31] reported positive associations with intake total foods consumption, fruits and vegetables, animal protein foods, white bread and snacks. Our findings are consistent with these previous studies but extend them by including a broader set of food groups and analysing results separately by sex, which offers a more detailed understanding of how Enjoyment of Food is expressed in preschool-aged children.

For the Food Responsiveness subscale, positive associations were found with vegetables, fruits and water, and negative associations with sugar and nuts, but only for boys. Previous studies have shown similar results, with higher Food Responsiveness levels associated with higher fruits and berries consumption [17, 31] and with higher meat consumption [32–34]. Carnel et al., also notes that the observed relationship is consistent with the subscale is objective, which is to take advantage of unhealthy cravings, including snacking, and eating more nutritious foods [31]. Food Responsiveness was also positively associated with the number of main meals per day [18].

Higher scores in the Desire to Drink subscale were positively associated with the consumption of water in both sexes and with processed meat, SSB and coffee and tea in boys, as well as dairy products in girls. Additionally, a higher score in Desire to Drink was negatively associated with nuts consumption in boys. This inverse association might reflect a preference for fluid over solid snacks in children with high Desire to Drink scores, possibly indicating lower interest in nutrient-dense, non-liquid foods such as nuts. The association between the Desire to Drink subscale and beverages consumption has been previously observed [35]. These findings on fluid intake corroborate another study assessing only fluid intake, which found that high Desire to Drink scores were associated with higher SSB intake [36]. These results suggest that a high Desire to Drink may reflect a general tendency toward increased beverage intake, regardless of the type. This trait could represent both a risk factor, when associated with sugar-sweetened beverages [36], and a potential opportunity for promoting healthier hydration habits in young children through targeted education and environmental strategies [37].

For Emotional Overeating different results were found according to sex. In boys, positive associations were found between this subscale with processed meat and prepared foods. In girls, positive associations were found with the whole grains group, whereas negative associations were observed with eggs, meat and processed meat. In both sexes, a negative association was found between Emotional Overeating and pulses consumption. As these results show positive associations with foods considered unhealthy, except for whole grains, and negative associations with nutrient-dense foods, this underlines the importance of taking foods consumption into account when analysing dietary behaviours [38]. These associations may reflect compensatory eating patterns, where emotional cues, particularly negative ones, lead to increased consumption of energy-dense foods. This is consistent with research showing that individuals with higher emotional eating tendencies eat more in response to negative affect, particularly calorie-dense snacks and processed items [39].

The integration of PCA and subsequent regression analysis provides a more nuanced understanding of the relationships between eating behaviours and dietary patterns in young children. This is especially relevant because the observed patterns were different in between boys and girls, underscoring the importance of considering sex-specific analysis in nutritional studies. Food-approach subscales, such as Enjoyment of Food and Food Responsiveness were also differentially associated with dietary patterns in boys and girls.

For Enjoyment of Food, boys showed a negative association with the Refined carbs pattern (characterised by high consumption of refined grains, sweets and sugar and low consumption of whole grains). Conversely, it was positively associated with the Meat & Refined Cereals pattern. In girls, Enjoyment of Food was positively associated with the Vegetables & Fish pattern. These associations suggest that children with higher Enjoyment of Food scores tend to follow more varied and nutrient-dense dietary patterns. While the association in boys with refined cereals and meats may reflect openness to a wider range of foods, the positive association in girls with a vegetable- and fish-rich pattern may indicate a tendency towards healthier preferences. This interpretation is supported by previous studies showing that children who enjoy eating are more likely to consume fruits and vegetables and have a broader food repertoire [40–42]. Although literature linking this subscale specifically to PCA-derived dietary patterns is lacking, our findings help fill this gap and suggest that Enjoyment of Food may be a valuable trait to reinforce through early intervention strategies.

Food Responsiveness, boys showed a negative association with the Refined carbs pattern. Similarly, in girls it was negatively associated with the Meat & Convenience pattern, which included high intakes of meat, tubers and processed foods. These findings may appear counterintuitive, as Food Responsiveness is generally considered a trait linked to increased intake. However, our results suggest that in this population, higher Food Responsiveness may be associated with more structured or regular eating, which might reduce the reliance on convenience or sugary foods. This interpretation aligns with previous work suggesting that the expression of appetite-related traits can vary depending on environmental context and food availability [40].

For Desire to Drink, boys showed a positive association with the Processed & SSB pattern (characterised by high intakes of processed meat, snacks, sugar-sweetened beverages and prepared foods). In girls, this subscale was positively associated with the Sugary foods pattern, including sugar, cocoa, coffee and tea. These results suggest that children with a higher Desire to Drink score may be more likely to consume beverages and foods high in sugar, particularly when they are part of broader less healthy eating patterns [43]. While this trait may reflect a general inclination toward fluid intake, it appears to also capture susceptibility to sweetened or palatable drinks, especially in boys. This finding reinforces the need to consider beverage-related behaviours in dietary assessments and interventions, particularly given the link between sugar-sweetened beverage intake and increased health risks in childhood [37, 44].

Finally, in relation to Emotional Overeating, it was negatively associated with the Nuts & Oils pattern, which included high intake of healthy fats and nutrient-dense plant-based foods, exclusively in boys. This may suggest that children who score high on Emotional Overeating are less likely to adhere to healthier, plant-based dietary patterns, possibly due to a preference for more palatable and convenience foods. This observation is consistent with prior research showing that emotional eaters often gravitate toward high-sugar, high-fat processed foods and are less responsive to nutrient-rich options [19, 39, 45]. These findings highlight the importance of incorporating emotional regulation and coping strategies into dietary counselling and behavioural interventions, particularly in boys.

Comparison of our results with the existing literature is not limited by a lack of studies on eating behaviours or dietary intake, but rather by the absence of research examining these associations using multivariate approaches such as PCA, particularly in preschool-aged children. This critical knowledge gap indicates the need for further studies to validate and extend our findings in the broader context of research on children’s diet and eating behaviours.

### Food-avoidant subscales

Concerning the food-avoidant subscales, the Food Fussiness and Satiety Responsiveness subscales showed a higher number of associations with food group intake and dietary patterns. In contrast, Slowness in Eating and Emotional Undereating showed a fewer associations, although all four traits were generally associated to lower intake of nutrient-dense food groups such as vegetables, fruits, whole grains and fish. It has been described that children who are fussier with food are less likely to consume healthy foods and more likely to consume energy-dense foods [46]. In addition, the Food Fussiness is the subscale that is more easily identified by parents when completing the questionnaire.

In this analysis, Food Fussiness subscale was consistently associated with reduced consumption of nutrient dense-foods. In both sexes, higher scores were negatively associated with intake of fish and seafood, vegetables, fruit and pulses. Additionally, this subscale was positively associated with consumption of sweets. Boys also showed a higher intake of sugar and cocoa, and a negative association with nuts and oils. On the other hand, girls showed positive associations with a processed meat and prepared foods, and negative associations with whole grains and oils. These results are in line with previous studies linking food fussiness to reduced vegetable intake [13, 32–34], and higher consumption of snacks [15, 18, 31].

Satiety Responsiveness also showed strong associations with dietary patterns. Children with higher scores tended to consume less vegetables, meat, fish, fruits, pulses, and whole grains, and more sweets and sugary foods. This pattern was evident in both sexes. While Satiety Responsiveness is commonly interpreted as a protective trait against overeating, our findings suggest that heightened sensitivity to satiety cues may reduce dietary variety, particularly when it results in refusal of meals or smaller portions of nutrient-dense foods. This is supported by Carnell et al. [31] who observed that children with high satiety responsiveness also consumed less meat, fruits, vegetables, snacks, and white bread.

Slowness in Eating was associated with lower intake of meat, fish and fruits in boys, and with higher intake of processed meat and lower intake of water in girls. Although fewer associations were observed, this trait may still influence food intake by altering mealtime dynamics, particularly if eating slowly leads to incomplete meals or parental interference. Previous studies have also found that slower eaters tend to consume less vegetables [33, 34].

Finally, on the Emotional undereating subscale, reflecting decreased appetite in response to emotional states, showed a negative association with pulses in both sexes. Boys also consumed more sugar and cocoa, while girls had lower intake of whole grains and water. These results contrast with a previous laboratory-based study, which found lower snack consumption and higher vegetable intake in children with higher emotional undereating scores [15]. This discrepancy is probably due to the more controlled observation of child mood in the cited study, as it was conducted in a laboratory setting under negative or neutral moos conditions.

In terms of dietary patterns, Food Fussiness and Satiety Responsiveness, showed different associations by sex. In boys, Food Fussiness was positively associated with the Refined carbs pattern ( characterised by high loadings for refined grains, sweets, sugar and cocoa, with a negative loading for whole grains), and negatively associated with both the Veg & fats pattern (rich in vegetables, nuts, and oils) and the Animal protein pattern (high in meat, fish, and refined cereals). In girls, Food Fussiness was negatively associated with the Veg-seafood-legumes pattern, composed of vegetables, fish, seafood and pulses. These associations are consistent with earlier studies linking food fussiness to the avoidance of nutrient-rich foods and the preference for highly palatable, processed items [12, 33, 34]. Although few studies have examined this trait in relation to PCA-derived dietary patterns, our findings suggest that food fussiness may limit adherence to balanced dietary profiles even from early childhood.

Satiety Responsiveness in boys was positively associated with the Refined carbs pattern and negatively associated with the Animal protein pattern. In girls, it showed a negative association with the Veg-seafood-legumes pattern. These results align with prior literature indicating that children with high satiety sensitivity often consume lower amounts of fruits, vegetables, and meat [17, 20]. Although this trait is frequently considered protective against overeating, our findings suggest that in some cases, heightened satiety responsiveness may also result in reduced intake of nutrient-dense foods, potentially affecting overall diet quality.

Finally, Slowness in Eating was negatively associated with the Animal protein pattern in boys, but no consistent associations with dietary patterns were found in girls. Previous studies have suggested that slower eating rates in children may be related to lower intake of main food groups, including vegetables and proteins [33, 34].

In complement to eating behaviours, it is important to understand appetite regulation that is a complex interaction of psychological, social, genetic and environmental factors [47]. This understanding is important to elucidate the mechanisms of these associations between eating behaviours and consumption of specific food groups. The literature suggests that certain eating behaviours, such as an under-response to internal satiety signals and an over-response to external food stimuli such as taste, smell, availability and emotions, are strongly associated with obesity [5, 48].

The sex-specific associations observed in this study highlight the importance of considering biological and environmental differences when examining eating behaviours in early childhood. These differences may reflect a combination of factors, including early hormonal and neurodevelopmental influences on appetite regulation, as described by Desai and Ross [6], who discussed how early-life nutrition may affect long-term appetite and metabolic outcomes. Cultural and social influences also play a key role, Parental feeding practices and expectations often differ based on the child’s sex, and gendered experiences during early life may contribute to the shaping of eating behaviours, particularly among girls [2, 4]. In our study, boys showed stronger associations between food-avoidant traits and intake of processed or sugary foods, while girls tended to show greater dietary restriction linked to emotional traits. These patterns suggest that intervention strategies may benefit from being sex-sensitive, addressing the unique behavioural tendencies and social environments experienced by boys and girls.

For parents and caregivers, understanding these behavioural profiles may support the development of more effective feeding approaches tailored to the child’s temperament. At the policy level, these findings can inform the design of public health campaigns and school-based nutrition programs that recognize sex-related behavioural variability. Future research should explore the underlying mechanisms of these sex-specific patterns, including genetic, psychological, and environmental pathways. In this regard, Herle et al. [19]. reviewed how eating behaviours mediate the expression of genetic susceptibility to obesity in children. Moreover, another study discussed how ultra-processed food preferences may interact with reward systems differently in boys and girls [43], further supporting the need for targeted strategies.

### Strengths and limitations

Some strengths and limitations in relation to this study should be highlighted. Firstly, the analysis of the relationship between eating behaviours and food and beverage consumption was conducted comprehensively, separating assessments by sex and covering 23 food groups. Despite it is a strength of this study, this made direct comparisons with other studies difficult, as they often analyse a significantly smaller number of food groups and rarely segmented by sex, and considering dietary patterns; therefore, comparisons were not possible due to lack of literature evaluating them in children. It is important to note that the study sample consisted of Spanish preschool children, limiting the generalisability of the results to other populations. Third, the majority of participants’ mothers had medium to high educational levels, suggesting that the results may not be fully representative for all education levels. Furthermore, we didn´t consider other influencing aspects such as parent´s behaviours and parenting models that are also related with children’s eating behaviours and foods intake. Among the strengths of this study, the significant sample size stands out, as well as the use of the Spanish version of the CEBQ, previously validated by us, with high reliability and internal validity. The analysis of the results, stratified by sex, highlights the differences and relevance of eating behaviours. These aspects consolidate this tool as a useful instrument for evaluating eating behaviours in this specific population.

## Conclusions

This study provides evidence on the associations between eating behaviours with food and beverage consumption in a sample of Spanish children. Although food-approach subscales are more likely to be associated with higher BMI or adiposity, some of these subscales, such as Enjoyment of Food and Food Responsiveness, were associated with high consumption of both nutrient-dense and energy- dense foods. While all food-avoidant subscales (Food Fussiness, Satiety Responsiveness, Slowness in Eating and Emotional Undereating) were associated with lower intake of nutrient-dense foods and higher intake of energy-dense foods such as snacks, sweets, sugar and cocoa, and processed meats. PCA revealed five distinct dietary patterns for both boys and girls. It is also important to highlight the differences between boys and girls, in terms of associations between the eating behaviours subscales and the different food and beverage groups. Further studies are needed to confirm these findings. These results have important implications not only for future research but also for public health strategies.

## Supplementary information

Below is the link to the electronic supplementary material.

Supplementary Material 1

Supplementary Material 2

Supplementary Material 3

## Data Availability

The datasets generated and analysed during the current study are not publicly available due to data regulations and for ethical reasons, considering that this information might compromise research participants’ acceptance because our participants only gave their consent for the use of their data by the original team of investigators. However, collaborations for data analyses can be requested by sending a letter to the CORALS steering Committee (estudiocoral@corals.es). The request will then be passed to all the members of the CORALS Steering Committee for deliberation.
