# Supplementary material for: Association between eating behaviours and food and beverage consumption in male and female children aged 3–6 years: The CORALS cohort
Source: Eur J Nutr. 2026 Jan 16;65(1):26. doi: 10.1007/s00394-025-03848-x (PMC12811333; doi:10.1007/s00394-025-03848-x)
Supplement: Supplementary file 1 — Supplementary Material 1 [file 394_2025_3848_MOESM1_ESM.docx]

| Food Group | Food in the FFQ |
| --- | --- |
| Dairy Products | Milk  Semi-skimmed milk  Skimmed milk  Lactose-free milk  Natural yoghurt without sugar  Natural sweetened yoghurt  Whole yoghurt  Low-fat yoghurt  Fermented milk  Milk curd  Cream cheese  Cheese  Fresh Cheese |
| Dairy desserts | Cream  Condensed milk  Milkshakes  Petit suitte  Custard  Ice cream |
| Meat | Chicken with skin  Chicken without skin  Veal  Pork  Lamb  Rabbit  Liver  Other entrails  Serrano Ham´  Ham |
| Processed meat | Processed meat  Pate  Hamburger  Bacon |
| Fish and Seafood | White Fish  Blue Fish  Salted Fish  Oysters  Squid  Shrimps  Surimi  Fish in oil |
| Vegetables | Vegetables type A  Vegetables type B  Garlic  Gazpacho  Mushrooms |
| Tubers | Baked Potato  French fries |
| Fruits | Citrus fruits  Banana  Fresh Fruits  Kiwi  Dates |
| Refined Grains | White bread  Tin loaf  Breadsticks  Unsweetened cereals  Rice  Pasta |
| Whole grains | Brown bread  Wholegrain cereals  Brown rice  Wholemeal pasta |
| Pulses | Pulses  Peas |
| Oils | Olive oil  Virgin olive oil  Olive pomace oil  Corn oil  Sunflower oil  Soy Oil  Oil blend  Mayonnaise  Margarine  Butter |
| Sweet | Sweetened cereals  Stuffed cereals  Chocolate  Biscuit  Chocolate Biscuit  Industrial pastries  Donuts  Cupcake  Cakes  Churros  Shortbread  Cocoa cream |
| Sugar and Cocoa | Sugar  Fruit in syrup  Honey and Jam  Sweets  Cocoa powder |
| Snacks | Snacks  Chips |
| Prepared Foods | Pizza  Croquettes  Soup |
| Sauces | Mustard  Ketchup  Salt  Iodised salt |
| Water | Tap water  Wottled water  Carbonated water |
| Sugar sweetened beverages | Fresh orange juice  Fresh fruit juice  Packaged fruit juice  Nectars  Flavoured water  Soft drinks with sugar  Sugar-free soft drinks  Isotonic semi-skimmed drinks  Energy drinks  Plant-based beverages |
| Coffee and tea | Coffee  Tea  Infusions |
|  |  |

|  |
| --- |
|  |
