# Supplementary material for: Association between eating behaviours and food and beverage consumption in male and female children aged 3–6 years: The CORALS cohort
Source: Eur J Nutr. 2026 Jan 16;65(1):26. doi: 10.1007/s00394-025-03848-x (PMC12811333; doi:10.1007/s00394-025-03848-x)
Supplement: Supplementary file 2 — Supplementary Material 2 [file 394_2025_3848_MOESM2_ESM.docx]

**Table S2.–** **Linear regression models for food groups intake according to CEBQ scales in children from the CORALS study (n=1407)**

| **CEBQ scales** | **Boys** | | **Girls** | |
| --- | --- | --- | --- | --- |
|  | **β (95% CI)** | **p- value** | **β (95% CI)** | **p-value** |
| **EF (Enjoyment of Food)** |  |  |  |  |
| Dairy products | -3.92 (-26.86 \| 19.01) | 0.737 | -11.88 (-32.97 \| 9.20) | 0.269 |
| Dairy desserts | -10.98 (-20.21 \| -1.76) | **0.020** | -3.16 (-12.41 \| 6.08) | 0.502 |
| Eggs | 1.44 (0.49 \| 2.39) | **0.003** | 1.80 (-0.22 \| 3.83) | 0.082 |
| Meat | 1.90 (-0.78 \| 4.58) | 0.165 | 0.61 (-1.67 \| 2.91) | 0.597 |
| Processed meat | -0.02 (-0.91 \| 0.86) | 0.956 | -0.80 (-1.85 \| 0.23) | 0.130 |
| Fish and seafood | 3.39 (1.48 \| 5.30) | **0.001** | 2.76 (0.51 \| 5.00) | **0.016** |
| Vegetables | 13.47 (8.62 \| 18.33) | **<0.001** | 10.46 (5.12 \| 15.79) | **<0.001** |
| Tubers | 0.87 (-1.43 \| 3.18) | 0.457 | -0.95 (-2.69 \| 0.77) | 0.279 |
| Fruits | 32.80 (21.33 \| 44.26) | **<0.001** | 13.84 (2.61 \| 25.07) | **0.016** |
| Nuts | 0.17 (-0.42 \| 0.76) | 0.574 | 0.29 (-0.33 \| 0.92) | 0.352 |
| Olives | 0.48 (0.00 \| 0.95) | **0.047** | 0.14 (-0.46 \| 0.76) | 0.638 |
| Refined grains | 1.29 (-1.80 \| 4.39) | 0.066 | -0.78 (-3.84 \| 2.27) | 0.614 |
| Whole grains | 1.59 (0.12 \| 3.06) | **0.033** | 3.07 (1.31 \| 4.84) | **<0.001** |
| Pulses | 1.17 (0.28 \| 2.06) | **0.010** | 1.52 (0.25 \| 2.79) | **0.028** |
| Oils | 0.12 (-1.22 \| 1.47) | 0.854 | 1.68 (0.30 \| 3.05) | **0.017** |
| Sweet | -5.84 (-8.93 \| -2.75) | **<0.001** | -3.47 (-6.38 \| -0.56) | **0.019** |
| Sugar and cocoa | -1.75 (-2.73 \| -0.77) | **<0.001** | -1.36(-1.06 \| 0.79) | 0.774 |
| Snacks | -0.91 (-1.93 \| 0.10) | 0.079 | -0.21 (-1.29 \| 0.85) | 0.694 |
| Prepared foods | 0.12 (-2.60 \| 2.84) | 0.203 | -3.44 (-6.31 \| -0.57) | **0.019** |
| Sauces | 0.00 (-0.16 \| 0.17) | 0.949 | -0.04 (-0.19 \| 0.10) | 0.547 |
| Water | 20.79 (-12.89 \| 54.48) | 0.387 | 47.73 (11.10 \| 84.37) | **0.011** |
| Sugar sweetened beverages | -2.65 (-17.18 \| 11.87) | 0.720 | -0.70 (-13.20 \| 8.82) | 0.912 |
| Coffee and tea | 1.68 (-0.73 \| 4.09) | 0.172 | -1.14 (-4.88 \| 2.60) | 0.550 |
| **FR (Food Responsiveness)** |  |  |  |  |
| Dairy products | -14.66 (-34.99 \| 5.67) | 0.157 | 1.36 (-17.27 \| 20.00) | 0.886 |
| Dairy desserts | -9.92 (-18.10 \| -1.73) | **0.018** | 0.46 (-7.70 \| 8.63) | 0.911 |
| Eggs | 0.52 (-0.31 \| 1.37) | 0.221 | 0.10 (-1.69 \| 1.89) | 0.911 |
| Meat | 0.86 (-1.51 \| 3.25) | 0.476 | -1.23 (-3.25 \| 0.79) | 0.234 |
| Processed meat | 0.67 (-0.11 \| 1.46) | 0.092 | -0.14 (-1.06 \| 0.78) | 0763 |
| Fish and seafood | 0.48 (-1.22 \| 2.20) | 0.575 | -0.65 (-2.63 \| 1.33) | 0.520 |
| Vegetables | 4.91 (0.52 \| 9.29) | **0.028** | 1.23 (-3.52 \| 5.99) | 0.610 |
| Tubers | -0.86 (-2.91 \| 1.18) | 0.408 | -0,53 (-2.06 \| 0.99) | 0.493 |
| Fruits | 18.99 (8.68 \| 29.30) | **<0.001** | 3.09 (-6.86 \|13.05) | 0.542 |
| Nuts | -0.26 (-0.36 \| 0.47) | 0.804 | -0.20 (-0.74 \| 0.34) | 0.464 |
| Olives | 0.05 (-0.27 \| 0.58) | 0.479 | -0.12 (-0.67 \| 0.42) | 0.650 |
| Refined grains | 1.47 (-1.27 \| 4.21) | 0.294 | -0.02 (-2.72 \| 2.68) | 0.987 |
| Whole grains | 0.57 (-0.73 \| 1.88) | 0.389 | 1.15 (-0.41 \| 2.72) | 0.148 |
| Pulses | 0.35 (-0.43 \| 1.14) | 0.380 | 0.52 (-0.60 \| 1.64) | 0.366 |
| Oils | -0.13 (-1.21 \| 1.18) | 0.983 | 0.75 (-0.46 \| 1.97) | 0.225 |
| Sweet | -1.41 (-4.18 \| 1.34) | 0.314 | -1.04 (-3.62 \| 1.53) | 0.426 |
| Sugar and cocoa | -1.15 (-2.02 \| -0.28) | **0.010** | -0.15 (-0.97 \| 0.66) | 0.716 |
| Snacks | 0.54 (-0.85 \| 9.63) | 0.907 | 0.93 (-0.01\| 1.88) | 0.053 |
| Prepared foods | 0.57 (-1.84 \| 2.99) | 0.640 | -2.32 (-4.86 \| 0.21) | 0.073 |
| Sauces | 0.55 (-0.93 \| 0.20) | 0.465 | -0.02 (-0.15 \| 0.10) | 0.678 |
| Water | 32.02 (2.18 \| 61.86) | **0.035** | 25.60 (-6.84 \| 58.05) | 0.122 |
| Sugar sweetened beverages | 7.07 (-5.81 \| 19.96) | 0.281 | 0.61 (-10.43 \| 11.65) | 0.913 |
| Coffee and tea | 1.61 (-0.53 \| 3.75) | 0.140 | -0.27 (-3.58 \| 3.03) | 0.871 |
| **DD (Desire to Drink)** |  |  |  |  |
| Dairy products | -0.64 (-20.95 \| 19.66) | 0.950 | 16.83 (-1.65\| 35.33) | 0.074 |
| Dairy desserts | -0.91 (-9.10 \| 7.28) | 0.827 | 4.81 (-3.30 \| 12.93) | 0.245 |
| Eggs | -0.25 (-1.09 \| 0.59) | 0.216 | -1.00 (-2.79 \| 0.78) | 0.270 |
| Meat | -0.53 (-2.91 \| 1.84) | 0.658 | -0.66 (-2.68 \| 1.35) | 0.518 |
| Processed meat | 0.99 (0.20 \| 1.78) | **0.013** | 0.44 (-0.47 \| 1.36) | 0.346 |
| Fish and seafood | -0.88 (-2.58 \| 0.82) | 0.311 | -0.12 (-2.09 \| 1.85) | 0.904 |
| Vegetables | -4.13 (-8.51 \| 0.24) | 0.064 | -0.34 ( -5.07 \| 4.39) | 0.888 |
| Tubers | -0.97 (-3.01 \| 1.07) | 0.351 | -0.15 (-1.67 \| 1.37) | 0.845 |
| Fruits | -4.40 (-14.78 \| 5.97) | 0.405 | -5.77 (-15.67 \| 4.11) | 0.252 |
| Nuts | -0.55 (-1.08 \| -0.03) | **0.037** | -0.22 (-0.77 \| 0.33) | 0.428 |
| Olives | 0.13 (-0.29 \| 0.55) | 0.470 | 0.04 (-0.49 \| 0.58) | 0.865 |
| Refined grains | 0.21 (-2.72 \| 2.76) | 0.988 | -2.60 (-5.28 \| 0.08) | 0.057 |
| Whole grains | -0.41 (-1.71 \| 0.89) | 0.536 | -1.15 (-2.71 \| 0.41) | 0.148 |
| Pulses | 0.51 (-0.27 \| 1.30) | 0.202 | -0.22 (-1.34 \| 0.89) | 0.695 |
| Oils | -1.04 (-2.23 \| 0.14) | 0.086 | -1.07 (-2.28 \| 0.13) | 0.082 |
| Sweet | 0.69 (-2.06 \| 3.45) | 0.623 | 0.92 (-1.64 \| 3.49) | 0.479 |
| Sugar and cocoa | -0.24 (-1.12 \| 0.62) | 0.576 | 0.21 (-0.60 \| 1.02) | 0.611 |
| Snacks | 0.77 (-0.13 \| 1.67) | 0.095 | 0.77 (-0.17 \| 1.71) | 0.109 |
| Prepared foods | 2.33 (-0.07 \| 4.74) | 0.057 | 1.70 (-0.82 \| 4.23) | 0.187 |
| Sauces | 0.03 (-0.11 \| 0.18) | 0.659 | -0.73 (-0.20 \| 0.58) | 0.274 |
| Water | 65.76 (36.31 \| 95.20) | **<0.001** | 64.78 (32.82 \| 96.74) | **<0.001** |
| Sugar sweetened beverages | 21.34 (8.58 \| 34.10) | **0.001** | 9.06 (-1.89 \| 20.02) | 0.105 |
| Coffee and tea | 3.25 (1.13 \| 5.38) | **0.003** | 2.76 (-0.51 \| 6.05) | 0.099 |
| **EOE (Emotional Overeating)** |  |  |  |  |
| Dairy products | -7.88 (-37.17 \| 21.40) | 0.656 | 6.63 (-20.50 \| 33.77) | 0.631 |
| Dairy desserts | -6.93 (-18.74 \| 4.87) | 0.249 | -1.41 (-13.32 \| 10.48) | 0.911 |
| Eggs | 1.19 (-0.02 \| 2.41) | 0.054 | -2.62 (-5.23 \| -0.18) | **0.048** |
| Meat | -1.97 (-5.40 \| 1.45) | 0.258 | -3.10 (-6.05 \| -0.16) | **0.039** |
| Processed meat | 1.29 (0.15 \| 2.43) | **0.026** | -1.53 (-2.87 \| -0.18) | **0.025** |
| Fish and seafood | -2.36 (-4.82 \| 0.09) | 0.059 | -1.34 (-4.23 \| 1.55) | 0.364 |
| Vegetables | -2.80 (-9.13 \| 3.52) | 0.384 | -2.22 (-9.15 \| 4.71) | 0.530 |
| Tubers | -0.47 (-3.43 \| 2.47) | 0.751 | -1.95 (-4.18 \| 0.26) | 0.085 |
| Fruits | -0.92 (-15.90 \| 14.04) | 0.903 | 0.15 (-14.40 \| 14.66) | 0.983 |
| Nuts | -0.45 (-1.21 \| 0.30) | 0.236 | 0.29 (-0.51 \| 1.10) | 0.480 |
| Olives | -0.32 (-0.92 \| 0.28) | 0.297 | -0.16 (-0.95 \| 0.62) | 0.680 |
| Refined grains | -0.26 (-4.21 \| 3.69) | 0.897 | -2.56 (-6.50 \| 1.36) | 0.200 |
| Whole grains | 0.72 (-1.15 \| 2.61) | 0.447 | 2.72 (0.44 \| 5.00) | **0.019** |
| Pulses | -1.13 (-2.46 \| -0.18) | **0.023** | -2.16 (-3.80 \| -0.53) | **0.009** |
| Oils | -1.26 (-2.98 \| 0.45) | 0.150 | -0.08 (-1.85 \| 1.69) | 0.930 |
| Sweet | 2.87 (-1.10 \| 6.85) | 0.156 | 3.26 (-0.48 \| 7.01) | 0.088 |
| Sugar and cocoa | 0.24 (-1.01 \| 1.51) | 0.698 | 0.44 (-0.75 \| 1.63) | 0.468 |
| Snacks | 0.04 (-1.26 \| 1.35) | 0.946 | 1.31 (-0.06 \| 2.69) | 0.620 |
| Prepared foods | 5.08 (1.62 \| 8.54) | **0.004** | -1.75 (-5.46 \| 1.95) | 0.354 |
| Sauces | 0.17 (-0.03 \| 0.38) | 0.102 | -0.09 (-0.28 \| 0.10) | 0.356 |
| Water | 26.23 (-16.78 \| 69.25) | 0.232 | -24.00 (-71.30 \| 23.30) | 0.320 |
| Sugar sweetened beverages | 8.16 (-10.37 \| 26.71) | 0.388 | 4.86 (-11.21 \| 20.94) | 0.552 |
| Coffee and tea | 1.24 (-1.84 \| 4.32) | 0.430 | 1.55 (-3.26 \| 6.37) | 0.526 |
| **FF (Food Fussiness)** |  |  |  |  |
| Dairy products | 8.69 (-12.48 \| 29.86) | 0.421 | 9.76 (-9.21 \| 28.80) | 0.312 |
| Dairy desserts | 7.66 (-0.86 \| 16.19) | 0.078 | 7.32 (-1.00 \| 15.64) | 0.085 |
| Eggs | - 0.84 (-1.72 \| 0.41) | 0.061 | -0.42 (-2.25 \| 1.41) | 0.652 |
| Meat | -1.03 (-3.51 \| 1.44) | 0.411 | -1.36 (-3.43 \| 0.70) | 0.195 |
| Processed meat | 0.21 (-0.61 \| 1.03) | 0.613 | 1.33 (0.39 \| 2.26) | **0.006** |
| Fish and seafood | - 3.70 (-5.46 \| -1.94) | **<0.001** | -5.03 (-7.02 \| -3.03) | **<0.001** |
| Vegetables | -17.41 (-21.80 \| -13.02) | **<0.001** | -17.65 (-22.33 \| -12.97) | **<0.001** |
| Tubers | -0.91 (-3.04 \| 1.22) | 0.403 | 0.42 (-1.13 \| 1.99) | 0.592 |
| Fruits | -28.89 (-39.50 \| -18.28) | **<0.001** | -24.44 (-34.45 \| -14.44) | **<0.001** |
| Nuts | -0.57 (-1.12 \| -0.02) | **0.040** | - 0.37 (-0.93 \| 0.19) | 0.199 |
| Olives | - 0.77 (-1.21 \| -0.34) | **<0.001** | -0.26 (-0.81 \| 0.29) | 0.197 |
| Refined grains | 0.36 (-2.49 \| 3.22) | 0.803 | 1.23 (-1.52 \| 3.98) | 0.381 |
| Whole grains | -0.49 (-1.85 \| 0.86) | 0.472 | -3.18 (-4.77 \| -1.60) | **<0.001** |
| Pulses | -1.06 (-1.89 \| -0.24) | **0.011** | -1.30 (-2.44 \|-0.15) | **0.026** |
| Oils | -0.33 (-1.58 \| 0.90) | 0.595 | -1.53 (-2.77 \| -0.29) | **0.015** |
| Sweet | 4.58 (1.73 \| 7.44) | **0.034** | 4.13 (1.51 \| 6.74) | **0.002** |
| Sugar and cocoa | 1.37 (0.46 \| 2.28) | **0.003** | -0.37 (-1.21 \| 0.46) | 0.382 |
| Snacks | 0.59 (-0.34\| 1.54) | 0.214 | 0.92 (-0.04 \| 1.88) | 0.620 |
| Prepared foods | -0.15 (-2.66 \| 2.36) | 0.907 | 3.50 (0.91 \| 6.09) | **0.008** |
| Sauces | -0.02 (-0.17 \| 0.12) | 0.756 | -0.00 (-0.14 \| 0.12) | 0.930 |
| Water | 22.14 (-8.94 \| 53.23) | 0.162 | -16.37 (-49.53 \| 16.78) | 0.333 |
| Sugar sweetened beverages | -2.00 (-15.41 \| 11.41) | 0.770 | 6.92 (-4.33 \| 18.18) | 0.227 |
| Coffee and tea | -1.75 (-3.98 \| 0.47) | 0.122 | 0.13 (-3.24 \| 3.51) | 0.937 |
| **SR (Satiety Responsiveness)** |  |  |  |  |
| Dairy products | 24.23 (-1.14 \| 49.61) | 0.061 | 11.66 (-10.30 \| 33.62) | 0.297 |
| Dairy desserts | 3.22 (-7.04 \| 13.48) | 0.538 | -1.82 (-11.46 \| 7.81) | 0.710 |
| Eggs | -0.50 (-1.11 \| 1.00) | 0.926 | -2.54 (-4.65 \| -0.43) | **0.018** |
| Meat | -3.13 (-6.10 \| -0.16) | **0.039** | -1.34 (-3.73 \| 1.04) | 0.271 |
| Processed meat | -0.60 (-1.58 \| 0.38) | 0.234 | 0.20 (-0.88 \| 1.29) | 0.716 |
| Fish and seafood | -2.26 (-4.82 \| -0.56) | **0.013** | -1.81 (-4.15 \| 0.52) | 0.129 |
| Vegetables | -11.29 (-16.73 \| -5.86) | **<0.001** | -7.04 (-12.63 \| -1.44) | **0.014** |
| Tubers | -0.30 (-2.87 \| 2.25) | 0.814 | -0.48 (-2.28 \| 1.32) | 0.601 |
| Fruits | -20.01 (-32.93 \| -7.09) | **0.002** | -7.04 (-18.77 \| 4.69) | 0.239 |
| Nuts | 0.12 (-0.53 \| 0.78) | 0.718 | -0.25 (-0.91 \| 0.39) | 0.438 |
| Olives | 0.35 (-0.17 \| 0.87) | 0.191 | -0.14 (-0.78 \| 0.49) | 0.663 |
| Refined grains | -2.25 (-5.68 \| 1.18) | 0.198 | -0.13 (-3.32 \| 3.05) | 0.933 |
| Whole grains | -0.03 (-1.66 \| 1.60) | 0.969 | -2.33 (-4.17 \| -0.48) | **0.013** |
| Pulses | -1.00 (-1.99 \| -0.01) | **0.047** | -0.61 (-1.94 \| 0.71) | 0.363 |
| Oils | -0.27 (-1.76 \| 1.22) | 0.723 | -1.15 (-2.59 \| 0.27) | 0.113 |
| Sweet | 2.67 (-0.78 \| 6.12) | 0.129 | 3.22 (0.18 \| 6.25) | **0.038** |
| Sugar and cocoa | 1.83 (0.74 \| 2.91) | **0.001** | 0.46 (-0.50 \| 1.43) | 0.343 |
| Snacks | 0.28 (-0.84 \| 1.42) | 0.618 | 0.39 (-0.72 \| 1.51) | 0.490 |
| Prepared foods | 0.16 (-2.86 \| 3.18) | 0.917 | 2.32 (-0.67 \| 5.32) | 0.128 |
| Sauces | 0.04 (-0.14 \| 0.22) | 0.647 | 0.10 (-0.05 \| 0.26) | 0.182 |
| Water | -25.31 (-62.66 \| 12.04) | 0.184 | -53.50 (-91.63 \| -15.38) | **0.006** |
| Sugar sweetened beverages | 1.17 (-14.94 \| 17.28) | 0.886 | 6.87 (-6.13 \| 19.88) | 0.300 |
| Coffee and tea | 2.03 (-0.63 \| 4.70) | 0.136 | 3.29 (-0.59 \| 7.19) | 0.097 |
| **LE (Slowness in eating)** |  |  |  |  |
| Dairy products | 8.97 (-12.00 \| 29.95) | 0.401 | 0.81 (-18.56 \| 20.20) | 0.934 |
| Dairy desserts | 5.24 (-3.22 \| 13.70) | 0.224 | -4.80 (-13.29 \| 3.68) | 0.267 |
| Eggs | -0.10 (-0.98 \| 0.76) | 0.809 | -0.71 (-2.58 \| 1.15) | 0.452 |
| Meat | -2.62 (-5.07 \| -0.17) | **0.036** | 0.14 (-1.96 \| 2.25) | 0.895 |
| Processed meat | -0.09 (-0.91 \| 0.71) | 0.812 | 0.99 (0.03 \| 1.95) | **0.042** |
| Fish and seafood | -2.09 (-3.85 \| -0.33) | **0.019** | -0.24 (-2.31 \| 1.82) | 0.815 |
| Vegetables | -1.85 (-6.38 \| 2.68) | 0.423 | -2.70 (-7.65 \| 2.24) | 0.284 |
| Tubers | 0.15 (-2.10 \| 2.13) | 0.989 | -0.37 (-1.96 \| 1.22) | 0.647 |
| Fruits | -15.53 (-26.19 \| -4.86) | **0.004** | -0.38 (-10.74 \| 9.97) | 0.942 |
| Nuts | 0.03 (-0.50 \| 0.57) | 0.900 | 0.02 (-0.55 \| 0.60) | 0.926 |
| Olives | 0.55 (0.12 \| 0.99) | **0.011** | 0.21 (-0.34 \| 0.78) | 0.449 |
| Refined grains | -1.61 (-4.44 \| 1.21) | 0.263 | -0.71 (-3.52 \| 2.09) | 0.619 |
| Whole grains | 0.40 (-0.94 \| 1.74) | 0.559 | -0.89 (-2.52 \| 0.74) | 0.285 |
| Pulses | -0.74 (-1.56 \| 0.06) | 0.073 | -0.68 (-1.85 \| 0.48) | 0.252 |
| Oils | 0.03 (-1.19 \| 1.27) | 0.953 | -0.56 (-1.82 \| 0.70) | 0.385 |
| Sweet | 2.09 (-0.75 \| 4.94) | 0.149 | 1.83 (-0.84 \| 4.51) | 0.179 |
| Sugar and cocoa | 0.13 (-0.77 \| 1.03) | 0.777 | -0.04 (-0.90 \| 0.80) | 0.912 |
| Snacks | 0.46 (-0.47 \| 1.39) | 0.332 | -0.25 (-1.24 \| 0.72) | 0.606 |
| Prepared foods | -1.09 (-3.58 \| 1.39) | 0.389 | 1.45 (-1.19 \| 4.10) | 0.281 |
| Sauces | 0.19 (-0.13 \| 0.17) | 0.808 | 0.57 (-0.07 \| 0.19) | 0.410 |
| Water | -8.64 (-39.50 \| 22.20) | 0.582 | -39.24 (-72.92 \| -5.57) | **0.022** |
| Sugar sweetened beverages | -4.91 (-18.20 \| 8.37) | 0.468 | 2.10 (-9.37 \| 13.58) | 0.719 |
| Coffee and tea | -0.52 (-2.73 \| 1.68) | 0.638 | 2.16 (-1.27 \| 5.60) | 0.218 |
| **EUE (Emotional Undereating)** |  |  |  |  |
| Dairy products | -2.72 (-23.15 \| 17.70) | 0.793 | 4.32 (-12.93 \| 21.57) | 0.623 |
| Dairy desserts | 3.80 (-4.43 \| 12.04) | 0.365 | 2.73 (-4.83 \| 10.29) | 0.479 |
| Eggs | 0.07 (-0.77 \| 0.92) | 0.868 | -1.23 (-2.89 \| 0.42) | 0.146 |
| Meat | -0.16 (-2.55 \| 2.22) | 0.893 | 0.33 (-1.54 \| 2.21) | 0.724 |
| Processed meat | -0.04 (-0.83 \| 0.75) | 0.913 | 0.12 (-0.73 \| 0.97) | 0.781 |
| Fish and seafood | -0.98 (-2.70 \| 0.73) | 0.260 | 1.33 (-0.50 \| 3.17) | 0.155 |
| Vegetables | -2.82 (-7.23 \| 1.58) | 0.209 | 0.67 (-3.73 \| 5.08) | 0.762 |
| Tubers | 0.16 (-1.96 \| 2.15) | 0.926 | -0.12 (-1.54 \| 1.29) | 0.860 |
| Fruits | -5.01 (-15.45 \| 5.42) | 0.346 | -1.63 (-10.86 \| 7.58) | 0.727 |
| Nuts | 0.16 (-0.36 \| 0.69) | 0.536 | 0.06 (-0.45 \| 0.58) | 0.798 |
| Olives | -0.05 (-0.47 \| 0.37) | 0.817 | -0.11 (-0.61 \| 0.38) | 0.655 |
| Refined grains | -1.56 (-4.32 \| 1.18) | 0.264 | 0.37 (-2.13 \| 2.87) | 0.771 |
| Whole grains | 0.41 (-0.89 \| 1.72) | 0.536 | -1.95 (-3.40 \| -0.50) | **0.008** |
| Pulses | -0.90 (-1.69 \| -0.10) | **0.026** | -1.09 (-2.13 \| -0.50) | **0.040** |
| Oils | 0.60 (-0.59 \| 1.80) | 0.322 | -0.25 (-1.38 \| 0.87) | 0.659 |
| Sweet | 1.23 (-1.54 \| 4.00) | 0.384 | -0.88 (-3.27 \| 1.50) | 0.468 |
| Sugar and cocoa | 0.99 (0.11 \| 1.87) | **0.026** | 0.41 (-0.34 \| 1.17) | 0.282 |
| Snacks | -0.30 (-1.21 \| 0.60) | 0.508 | -0.00 (-0.88 \| 0.87) | 0.988 |
| Prepared foods | 0.93 (-1.49 \| 3.35) | 0.451 | 3.40 (1.05 \| 5.74) | **0.005** |
| Sauces | -0.03 (-0.18 \| 0.11) | 0.669 | 0.05 (-0.06 \| 0.17) | 0.385 |
| Water | -25.36 (-55.34 \| 4.60) | 0.970 | -35.67 (-65.65 \| -5.69) | **0.020** |
| Sugar sweetened beverages | -3.46 (-16.40 \| 9.47) | 0.599 | 3.66 (-6.56 \| 13.88) | 0.482 |
| Coffee and tea | 0.13 (-2.01 \| 2.28) | 0.904 | -0.15 (-3.22 \| 2.90) | 0.920 |

CEBQ: Child Eating Behavior Questionnaire. CI= Confidence Intervals

Bold letters show significant (p < 0.05). *Model adjusted for maternal education, age, center and energy intake.
