# Supplementary material for: Association between eating behaviours and food and beverage consumption in male and female children aged 3–6 years: The CORALS cohort
Source: Eur J Nutr. 2026 Jan 16;65(1):26. doi: 10.1007/s00394-025-03848-x (PMC12811333; doi:10.1007/s00394-025-03848-x)
Supplement: Supplementary file 3 — Supplementary Material 3 [file 394_2025_3848_MOESM3_ESM.docx]

**Table S3.–** **Linear regression models for dietary patterns according to CEBQ scales in children from the CORALS study (n=1407)**

| **CEBQ scales** | **Boys** | | **Girls** | |
| --- | --- | --- | --- | --- |
|  | **β (95% CI)** | **p- value** | **β (95% CI)** | **p-value** |
| **EF (Enjoyment of Food)** |  |  |  |  |
| Dietary patterns 1 | -0.038 (-0.115 \| 0.039) | 0.331 | 0.207 (0.119 \| 0.295) | **<0.001** |
| Dietary patterns 2 | -0.206 (-0.287 \| -0.125) | **<0.001** | -0.040 (-0.128 \| 0.047) | 0.367 |
| Dietary patterns 3 | 0.163 (0.073 \| 0.252) | **<0.001** | -0.007 (-0.095 \| 0.081) | 0.881 |
| Dietary patterns 4 | -0.026 (-0.104 \| 0.052) | 0.520 | 0.068 (-0.021 \| 0.157) | 0.135 |
| Dietary patterns 5 | 0.064 (-0.018 \| 0.146) | 0.123 | -0.018 (-0.100 \| 0.065) | 0.674 |
| **FR (Food Responsiveness)** |  |  |  |  |
| Dietary patterns 1 | 0.024 (-0.044 \| 0.093) | 0.483 | 0.038 (-0.041\| 0.117) | 0.343 |
| Dietary patterns 2 | -0.089 (-0.162 \| -0.016) | **0.017** | -0.075 (-0.152 \| 0.002) | 0.058 |
| Dietary patterns 3 | 0.072 (-0.008 \| 0.152) | 0.077 | 0.014 (-0.064 \| 0.092) | 0.727 |
| Dietary patterns 4 | -0.069 (-0.138 \| 0.001) | 0.052 | 0.026 (-0.053 \| 0.105) | 0.519 |
| Dietary patterns 5 | -0.015 (-0.087 \| 0.058) | 0.695 | -0.004 (-0.076 \| 0.069) | 0.919 |
| **DD (Desire to Drink)** |  |  |  |  |
| Dietary patterns 1 | 0.102 (0.034 \| 0.170) | **0.003** | -0.052 (-0.131\| 0.026) | 0.190 |
| Dietary patterns 2 | -0.035 (-0.108 \| 0.038) | 0.351 | -0.003 (-0.080 \| 0.075) | 0.947 |
| Dietary patterns 3 | -0.003 (-0.083 \| 0.077) | 0.936 | 0.094 (0.017 \| 0.171) | **0.016** |
| Dietary patterns 4 | -0.011 (-0.059 \| 0.080) | 0.765 | -0.013 (-0.091 \| 0.065) | 0.742 |
| Dietary patterns 5 | -0.117 (-0.189 \| -0.045) | **0.002** | -0.033 (-0.105 \| 0.039) | 0.374 |
| **EOE (Emotional Overeating)** |  |  |  |  |
| Dietary patterns 1 | 0.071 (-0.028 \| 0.169) | 0.158 | -0.053 (-0.168 \| 0.062) | 0.364 |
| Dietary patterns 2 | 0.037 (-0.068\| 0.143) | 0.487 | -0.112 (-0.225 \| 0.000) | 0.051 |
| Dietary patterns 3 | -0.070 (-0.186 \| 0.045) | 0.232 | 0.080 (-0.033 \| 0.193) | 0.166 |
| Dietary patterns 4 | -0.054 (-0.154 \| 0.045) | 0.283 | -0.055 (-0.170 \| 0.060) | 0.347 |
| Dietary patterns 5 | -0.152 (-0.252 \| -0.048) | **0.004** | 0.081 (-0.025 \| 0.186) | 0.134 |
| **FF (Food Fussiness)** |  |  |  |  |
| Dietary patterns 1 | -0.006 (-0.077 \| 0.065) | 0.868 | -0.309 (-0.386 \| -0.231) | **<0.001** |
| Dietary patterns 2 | 0.187 (0.112 \| 0.262) | **<0.001** | 0.000 (-0.080 \| 0.079) | 0.994 |
| Dietary patterns 3 | - 0.141 (-0.224 \| 0.059) | **0.001** | -0.002 (-0.081 \| 0.078) | 0.967 |
| Dietary patterns 4 | 0.024 (-0.048 \| 0.096) | 0.509 | -0.039 (-0.119 \| 0.041) | 0.339 |
| Dietary patterns 5 | -0.140 (-0.215 \| -0.065) | **<0.001** | 0.016 (-0.058 \| 0.090) | 0.676 |
| **SR (Satiety Responsiveness)** |  |  |  |  |
| Dietary patterns 1 | 0.026 (-0.059 \| 0.112) | 0.544 | -0.124 (-0.216 \| -0.031) | **0.009** |
| Dietary patterns 2 | 0.126 (0.035\| 0.217) | **0.007** | -0.008 (-0.100 \| 0.083) | 0.862 |
| Dietary patterns 3 | -0.185 (-0.284 \| -0.086) | **<0.001** | 0.062 (-0.030 \| 0.153) | 0.187 |
| Dietary patterns 4 | -0.027 (-0.060 \| -0.113) | 0.541 | -0.037 (-0.130 \| 0.056) | 0.437 |
| Dietary patterns 5 | -0.038 (-0.129 \| 0.053) | 0.409 | 0.006 (-0.080 \| 0.091) | 0.896 |
| **LE (Slowness in eating)** |  |  |  |  |
| Dietary patterns 1 | 0.030 (-0.040 \| 0.101) | 0.398 | -0.059 (-0.141 \| 0.023) | 0.160 |
| Dietary patterns 2 | 0.022 (-0.053 \| 0.098) | 0.560 | 0.006 (-0.075 \| 0.087) | 0.880 |
| Dietary patterns 3 | -0.135 (-0.217 \| -0.053) | **0.001** | 0.006 (-0.075 \| 0.087) | 0.884 |
| Dietary patterns 4 | 0.023 (-0.048 \| 0.094) | 0.528 | -0.010 (-0.092 \| 0.072) | 0.806 |
| Dietary patterns 5 | 0.021 (-0.054 \| 0.096) | 0.585 | 0.037 (-0.038 \| 0.113) | 0.336 |
| **EUE (Emotional Undereating)** |  |  |  |  |
| Dietary patterns 1 | 0.009 (-0.060 \| 0.078) | 0.801 | -0.021 (-0.094 \| 0.052) | 0.573 |
| Dietary patterns 2 | 0.048 (-0.025 \| 0.121) | 0.200 | 0.050 (-0.022 \| 0.122) | 0.173 |
| Dietary patterns 3 | -0.048 (-0.128 \| 0.033) | 0.244 | -0.023 (-0.095 \| 0.049) | 0.532 |
| Dietary patterns 4 | -0.014 (-0.084 \| 0.055) | 0.689 | -0.028 (-0.101 \| 0.045) | 0.453 |
| Dietary patterns 5 | -0.006 (-0.079 \| 0.067) | 0.867 | -0.001 (-0.069 \| 0.066) | 0.970 |

CEBQ: Child Eating Behavior Questionnaire. CI= Confidence Intervals

Bold letters show significant (p < 0.05). *Model adjusted for maternal education, age, center and energy intake.
